# Systematic review of pre-clinical therapies for post-operative atrial fibrillation

**DOI:** 10.1371/journal.pone.0241643

**Published:** 2020-11-04

**Authors:** Chanhee Seo, Connor Michie, Benjamin Hibbert, Darryl R. Davis

**Affiliations:** Department of Medicine, Division of Cardiology, University of Ottawa Heart Institute, Ottawa, Canada; The Open University, UNITED KINGDOM

## Abstract

**Background:**

Post-operative atrial fibrillation (POAF) is a frequent cardiothoracic surgery complication that increases hospital stay, mortality and costs. Despite decades of research, there has been no systematic overview and meta-analysis of preclinical therapies for POAF in animal models.

**Methods:**

We performed a systematic search of MEDLINE and EMBASE from their inception through September 2020 to determine the effect of preclinical POAF therapies on primary efficacy outcomes using a prospectively registered protocol (CRD42019155649). Bias was assessed using the SYRCLE tool and CAMARADES checklist.

**Results:**

Within the 26 studies that fulfilled our inclusion criteria, we identified 4 prevention strategies including biological (n = 5), dietary (n = 2), substrate modification (n = 2), and pharmacological (n = 17) interventions targeting atrial substrate, cellular electrophysiology or inflammation. Only one study altered more than 1 pathophysiological mechanism. 73% comprised multiple doses of systemic therapies. Large animal models were used in 81% of the studies. Preclinical therapies altogether attenuated atrial fibrosis (SMD -2.09; 95% confidence interval [CI] -2.95 to -1.22; *p* < 0.00001; *I*^*2*^ = 47%), AF inducibility (RR 0.40; 95% CI 0.21 to 0.79; *p* = 0.008; *I*^*2*^ = 39%), and AF duration (SMD -2.19; 95% CI -3.05 to -1.32; *p* < 0.00001; *I*^*2*^ = 50%). However, all the criteria needed to evaluate the risk of bias was unclear for many outcomes and only few interventions were independently validated by more than 1 research group.

**Conclusion:**

Treatments with therapies targeting atrial substrate, cellular electrophysiology or inflammation reduced POAF in preclinical animal models compared to controls. Improving the quality of outcome reporting, independently validating promising approaches and targeting complimentary drivers of POAF are promising means to improve the clinical translation of novel therapies for this highly prevalent and clinically meaningful disease.

## Introduction

Post-operative atrial fibrillation (POAF) is a commonplace arrhythmia seen in a third of patients after coronary artery bypass grafting and almost half of patients after valve repair/replacement [1–3]. Albeit often transient, the impact of POAF on surgical outcomes is significant as it portends a 2-fold increase in mortality, greater hospital resource utilization and increased costs [4, 5]. Emerging evidence has shown that POAF arises from a combination of pre-existing cardiomyopathic changes in the atria, surgical-induced changes in atrial substrate and post-operative insults (such as inflammation, altered neural regulation and oxidative stress) [6–8]. These electrical and structural changes increase AF vulnerability by creating a pro-fibrillatory substrate while altered calcium handling increases the risk for delayed afterdepolarizations and the stimulation of ectopic atrial beats that trigger the arrhythmia [9–11].

Unfortunately, many of the standard therapies for paroxysmal AF have a very limited ability to prevent POAF. Once POAF occurs, routine rhythm control is not recommended as standard rhythm-control therapies are often ineffective or limited by off target side effects. As such, a number of dietary, interventional, molecular and pharmacologic agents that alter the drivers or substrate regulating POAF have been studied in preclinical models to identify an effective prophylactic strategy. The purpose of this systematic review is to provide a comprehensive overview of all preventative POAF studies in animal models reporting functional outcomes with an evaluation of the potential mechanisms, study design and bias. From this analysis, we identify promising strategies that positively influence post-surgical AF outcomes to provide recommendations for future pre-clinical and clinical trials.

## Methods

### Search strategy

We performed an electronic literature search of the Medical Literature Analysis and Retrieval System Online (MEDLINE) and the Excerpta Medica dataBASE (EMBASE) from database inception to September 2020 using methods prospectively registered in the International Prospective Register of Systemic Reviews (PROSPERO, CRD42019155649). To maximize the sensitivity of the search strategy, we combined the terms: “animal experimentation,” “postoperative period,” and “atrial fibrillation” or any of their synonyms as either MeSH terms or key words (S1 Table in S1 File). The search strategy was designed to capture all animal studies relevant to the study question as previously described [12]. In addition, a manual screening of the bibliographies of all retrieved articles was performed to enable a broad evaluation of the current literature.

### Study selection

Papers were independently screened by two investigators (CS and CM) in the title-abstract and full-text screen using the predefined inclusion and exclusion criteria described below. Before formally commencing the screening process, a calibration test using 10 randomly retrieved articles was executed to ensure high inter-rater validity. When no consensus on inclusion was met, a third investigator was consulted. Papers were included if they (1) reported efficacy outcomes of preclinical therapies for POAF, and (2) used animal models of postoperative atrial fibrillation (i.e., sterile pericarditis, atriotomy, pericardiotomy) that mimic the inflammatory state seen in postoperative setting [13, 14]. We included both single-arm studies, in which the effect of intervention was measured before and after administration, and double-arm studies, in which parallel intervention and control groups were measured. Papers were excluded if they (1) included human population (i.e., human clinical trials), (2) assessed efficacy outcomes through *in vitro* or *ex vivo* studies, (3) used irrelevant animal models of POAF (e.g., vagal induction, electrical pacing only), (4) studied downstream POAF reduction strategies (e.g., cardioversion, ablation), and (5) focused on other cardiac arrhythmias (e.g., atrial flutter, ventricular arrhythmia). We also excluded case-reports, review articles, grey literature, unpublished articles, and studies that were not published in the English language.

### Data extraction and analysis

A standardized data abstraction table was created *a priori* by the review team to extract all relevant data from full-text articles. Two reviewers (CS and CM) independently extracted the data and compared the results for verification. Extracted data included study characteristics (e.g., sample size, randomization, study design, blinding of outcome analysis, endpoint), intervention description (e.g., type and frequency of intervention, mode and dosage of administration), animal model (e.g., species, POAF model, base characteristics), and primary (atrial fibrosis, AF inducibility, termination, duration) and secondary (e.g., electrophysiological measurements, conduction mapping) outcomes. Outcomes reported only in graphical format were extracted using the web-based software WebPlotDigitizer (Version 4.3; https://automeris.io/WebPlotDigitizer/). The SYstematic Review Centre for Laboratory animal Experimentation (SYRCLE) risk of bias tool was used to assess study bias [15]. Each criteria of the SYRCLE tool (selection, performance, detection, attrition, reporting, other bias) was assessed by two independent investigators (CS and CM) and a value of low, high, or unclear risk of bias was assigned for each included study. Quality assessment was performed using the Collaborative Approach to Meta-Analysis and Review of Animal Data from Experimental Studies (CAMARADES) checklist [16]. Each study was assessed independently by the same two investigators and a global quality assessment value of poor, average, or good quality was determined as per the checklist. Any disagreements were resolved by discussion and consensus.

Data are expressed as mean ± SD unless otherwise specified. Significance level was determined *a priori* to *p* < 0.05. To account for heterogeneity of treatment effect between the reviewed studies, random effects meta-analyses were performed using the DerSimonian and Laird model for dichotomous variables (e.g., AF inducibility, AF termination) and random effects inverse variance meta-analysis for continuous variables (e.g., atrial fibrosis, AF duration) before and after the treatment to determine the overall effect size of each outcome [17]. Dichotomous variables were reported as risk ratios (RR), and continuous variables as standardized mean differences (SMD) due to the considerable differences in the working definition of AF and measurement of outcomes in the included studies. In cases where the risk ratio could not be calculated due to zero events in one or both groups, a continuity correction was performed by assigning a fixed value of 0.5 to all cells in the 2x2 table to avoid computational errors [18]. *Z* test was performed to determine the *P-*value for the overall effect of the comparisons. Heterogeneity of effect sizes was assessed using the Cochrane *I*^2^ statistic with the following thresholds: 0–40% (low heterogeneity), 30–60% (moderate), 50–90% (substantial), and 75–100% (considerable) [18]. Subgroup analysis was performed based on different study characteristics, such as the size and type of animal models, and therapeutic strategies if there was considerable heterogeneity (75–100%) to elucidate the source of heterogeneity. Furthermore, in case of considerable heterogeneity, sensitivity analysis was also performed by eliminating a single study at a time to elicit the impact of the study on the overall result. To evaluate potential publication bias, funnel plots were produced to assess the symmetry in plotted values [19]. The meta-analysis was performed using the Cochrane Collaboration’s Review Manager statistical software (RevMan, 5.4).

## Results

**Fig 1:**
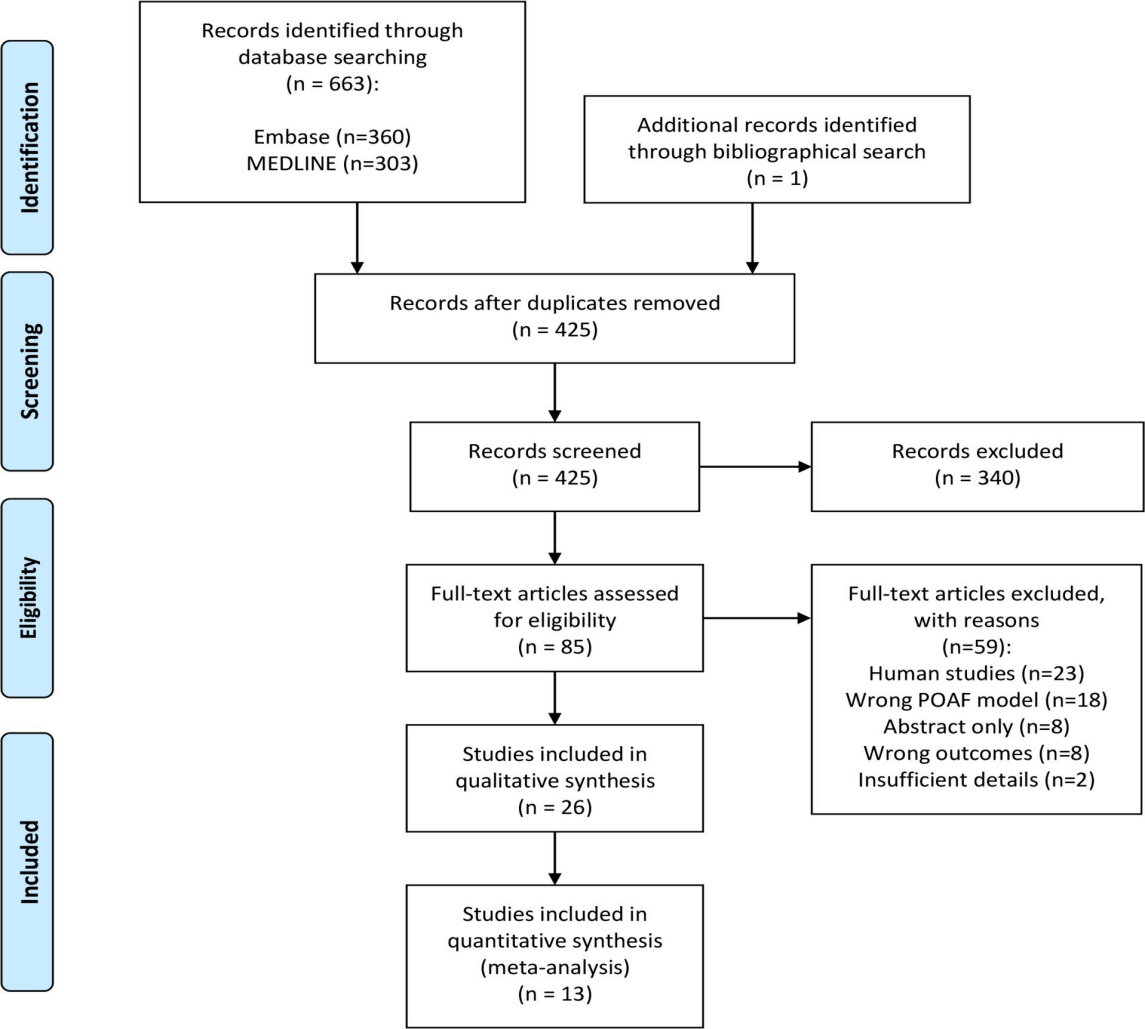
PRISMA flow diagram outlining the systematic search performed on September 03, 2020.

Fig 1 illustrates the study selection process in a PRISMA flow diagram. The search strategy identified a total of 360 and 303 citations from EMBASE and MEDLINE, respectively. One additional citation was identified from a manual bibliographical search that was performed during title-abstract screening [20]. After removing duplicate search results and screening for relevant title and abstract, 85 articles were identified for full-text screening. Of these, 59 studies were further excluded as they did not meet the inclusion criteria. In total, 26 studies were included in this review [20–45].

Despite decades of clinical observation and reports on POAF, studies on pre-clinical therapies gained increasing attention at the turn of the century with only one study dating back to 1993 [21]. As shown in Table 1, pre-clinical therapies could be largely grouped into four themes: biologics (5 out of 26 studies) [29, 37, 38, 42, 44], dietary modification (2 studies) [33, 34], electrical substrate alteration (1 study) [22], anatomical substrate alteration (1 study) [40], and pharmacologic approaches (17 studies) [20, 21, 23–28, 30–32, 35, 36, 39, 41, 43, 45]. Of these, three of the four studies using methylprednisolone were published by one laboratory [26, 35, 43] and both studies using vanoxerine were published by a second group [30, 32]. Early publications largely focused on pharmacologic means of reducing POAF, while recent publications showed more diversified approaches using dietary molecules and biologic targets. Twenty-one of the 26 studies were carried out in large animal models (e.g., canine, swine, goat) while small animal models (e.g., rat, rabbit) were only used in biologic therapy studies and two pharmacologic studies [20, 45]. Most studies performed either induction of sterile pericarditis during an open-heart surgery (19 studies) or atriotomy (5 studies) as pre-treatments to model POAF in the animal model. Of note, only two studies used pericardiotomy alone to model POAF, both of which were performed in rabbits [20, 44].

**Table 1:** Strategies for reducing post-operative atrial fibrillation in animal models.

Reference	Treatment	Method	Species	Animal model
**Biologics**				
Rossman et al., 2009 [29]	GAP-134 (gap junction modifier)	IV administration	Dog	Induction of SP
Fu et al., 2015 [37]	Anti-rat IL-17 mAb	IP injection	Rat	Induction of SP
Sadrpour et al., 2015 [38]	M-II (K201 metabolite)	IV administration	Dog	Induction of SP
Huang et al., 2016 [42]	S3I-201 (selective STAT3 inhibitor) & antagomir-21	IP injection & Plasmid IM injection	Rat	Induction of SP
Chang et al., 2018 [44]	MPT0E014 (HDAC inhibitor)	IV administration	Rabbit	Pericardiotomy
**Dietary modification**				
Mayyas et al., 2011 [33]	n-3 PUFA (EPA+DHA)	Diet supplement	Dog	Atriotomy
Zhang et al., 2011 [34]	n-3 PUFA (EPA+DHA)	Oral administration	Dog	Induction of SP
**Substrate alteration**				
Becker et al., 2002 [22]	Multisite & septal atrial pacing	Constant pacing from HRA, LRA, HLA, LLA and septal electrodes	Dog	Induction of SP
Yang et al., 2015 [40]	Modification of RA free wall incision	Modified atriotomy incisional line extending from IVC to TA	Pig	Atriotomy
**Pharmacologic**				
Shimizu et al., 1993 [21]	E-4031 (class III antiarrhythmic agent)	IV administration	Dog	Induction of SP
Kumagai et al., 2003 [23]	JTV-519 (K201; RyR-channel inhibitor)	IV administration	Dog	Induction of SP
Goldstein et al., 2004 [24]	AZD7009 (K^+^ & Na^+^ channel blocker)	IV administration	Dog	Induction of SP
Kumagai et al., 2004 [25]	Atorvastatin	Oral administration	Dog	Induction of SP
Ishii et al., 2005 [26]	Methylprednisolone	NS (2 mg/kg per day)	Dog	Atriotomy
Tselentakis et al., 2006 [27]	Ibuprofen & methylprednisolone	Topical atrial application	Dog	Induction of SP
Goldstein et al., 2008 [28]	Prednisone	Oral administration	Dog	Induction of SP
Matsumoto et al., 2010 [30]	Vanoxerine	IV administration	Dog	Induction of SP
Yoo et al., 2010 [31]	Triamcinolone	Triamcinolone + fibrin applied to atria	Dog	Induction of SP
Cakulev et al., 2011 [32]	Vanoxerine	Oral administration	Dog	Induction of SP
Schuessler et al., 2012 [35]	Methylprednisolone	NS (2 mg/kg per day)	Dog	Atriotomy
Bhimani et al., 2014 [36]	Ranolazine	IV administration	Dog	Induction of SP
Zhang et al., 2015 [39]	Atorvastatin	Oral administration	Goat	Induction of SP
Schwartzman et al., 2016 [41]	Amiodarone	Attachment of PBM on atrial epicardial surface	Pig	Induction of SP
Robinson et al., 2016 [20]	PPX[AMIO, DEX]	Attachment of parylene-C film fixed on pericardium	Rabbit	Pericardiotomy
Ishii et al., 2017 [43]	Methylprednisolone	NS (2 mg/kg per day)	Dog	Atriotomy
Wu et al., 2020 [45]	Colchicine	Oral administration	Rat	Induction of SP

DHA = docosahexaenoic acid; EPA = eicosapentaenoic acid; HDAC = histone deacetylase; HLA = high left atrium; HRA = high right atrium; IL-17 = interleukin 17; IM = intramyocardial; IP = intraperitoneal; IV = intravenous; IVC = inferior vena cava; LLA = low left atrium; LRA = low right atrium; mAb = monoclonal antibody; miR = microRNA; NS = not specified; PBM = Plasma-Based Amiodarone-Impregnated Material; POAF = postoperative atrial fibrillation; PPX[AMIO, DEX] = amiodarone- and dexamethasone-loaded parylene-C film; PUFA = polyunsaturated fatty acid; RA = right atrium; SP = sterile pericarditis; RyR = ryanodine receptor; STAT3 = signal transducer and activator of transcription 3; TA = tricuspid annulus.

S2 Table in S1 File presents descriptions of the 26 included studies. Experimental groups ranged between 5 and 12 animals, with only 3 studies reporting more than 10 animals in both experimental and control groups. Eight studies were single-arm studies in which the effect of a therapeutic intervention was evaluated at baseline and after administration for all animals used in the study. Primary outcomes typically evaluated short-term (i.e., less than a week) effects of treatments in POAF characteristics (23 studies), in keeping with the natural prevalence of POAF in hospitalized patients.

As shown in Table 2, 18 interventions reduced AF inducibility [20, 22–24, 26, 27, 29, 30, 32–34, 37, 41–43, 45], 1 intervention increased AF inducibility [28], and 2 interventions had no effect [31, 39]. Six studies demonstrated an intervention that increased spontaneous termination of AF [21, 24, 30, 32, 36, 38]. Of the 13 studies that explored the effect of an intervention on AF duration, 11 found a significant reduction [20, 25, 26, 29, 34, 37, 39, 41–43, 45] while 2 failed to demonstrate any effect [22, 31]. Of the 10 studies exploring effects on atrial remodelling, 9 interventions were found to significantly attenuate atrial remodelling [20, 34, 37, 39–42, 44, 45] while 1 study identified an intervention that adversely impacted atrial remodeling but, given the nature of the intervention (i.e., extensive atriotomy incisions), such an effect was expected [40]. Among the non-substrate interventions, only 6 were performed as a single treatment as opposed to multiple systematic treatments. These included miR-21 injection into the left atrial wall [42], plasma-based amiodarone-impregnated material gel on the atrial surfaces [41], ibuprofen and methylprednisolone powder on the atrial surfaces [27], triamcinolone spray on the epicardium [31], and dexamethasone + amiodarone releasing nano-structured parylene-C film on the epicardium [20].

**Table 2:** Study outcomes reported.

● single study suggesting efficacy	Reduced AF inducibility	Enhanced AF termination	Reduced AF duration	Reduced AT inducibility	Reduced spontaneous AT	Enhanced AFL termination	Reduced atrial fibrosis	Reduced atrial inflammation	Prolonged AERP/ARP	Reduced heart rate	Reduced conduction inhomogeneity	Reduced conduction time	Increased capture threshold	Large animal model	Number of independent labs
○ single study suggesting no effect															
● single study suggesting opposite effect															
● multiple studies supporting efficacy															
○ multiple studies supporting no effect															
? multiple studies demonstrating conflicting results															
Reference															
MPT0E014 (HDAC inhibitor) [44]				●			●			○					1
S3I-201 (selective STAT3 inhibitor) [42]	●		●				●	●			●				1
antagomir-21 [42]	●						●	●			●				1
Anti-IL-17A mAb [37]	●		●				●	●	●						1
M-II (K201 metabolite) [38]		●							●			●	●	●	1
GAP-134 (gap junction modifier) [29]	●		●	●					○	○		●		●	1
n-3 PUFA (EPA+DHA) [33, 34]	●		●				●	●	●	●		●		●	2
Extended atriotomy incision [40]	○			●			●							●	1
Multisite & septal atrial pacing [22]	●		○											●	1
Amiodarone [41]	●		●				●	●	●			○		●	1
Triamcinolone [31]	○		○		●			●	○					●	1
Methylprednisolone [26, 27, 35, 43]	●		●					●	○	?	●	○		●	2
Prednisone [28]	●			●				●			●		●	●	1
Ibuprofen [27]	●								○	○		○		●	1
Atorvastatin [25, 39]	○		●				●	●	●			●		●	2
Vanoxerine [30, 32]	●	●		●		●			●			○	?	●	2
Ranolazine [36]		●				●			●			●	●	●	1
AZD7009 (K^+^ & Na^+^ channel blocker) [24]	●	●		●		●			●			●	●	●	1
JTV-519 (K201; RyR-channel inhibitor) [23]	●								●			○		●	1
E-4031 (class III antiarrhythmic agent) [21]		●							●			○		●	1
Colchicine [45]	●		●				●	●	○	●	●	○			1
PPX[AMIO, DEX] [20]	●		●				●	●							1

AERP = atrial effective refractory period; AF = atrial fibrillation; AFL = atrial flutter; ARP = atrial nodal refractory period; AT = atrial tachyarrhythmia; DHA = docosahexaenoic acid; EPA = eicosapentaenoic acid; HDAC = histone deacetylase; IL-17 = interleukin 17; PPX[AMIO, DEX] = amiodarone- and dexamethasone-loaded parylene-C film; PUFA = polyunsaturated fatty acid; RyR = ryanodine receptor; STAT3 = signal transducer and activator of transcription 3.

**Fig 2:**
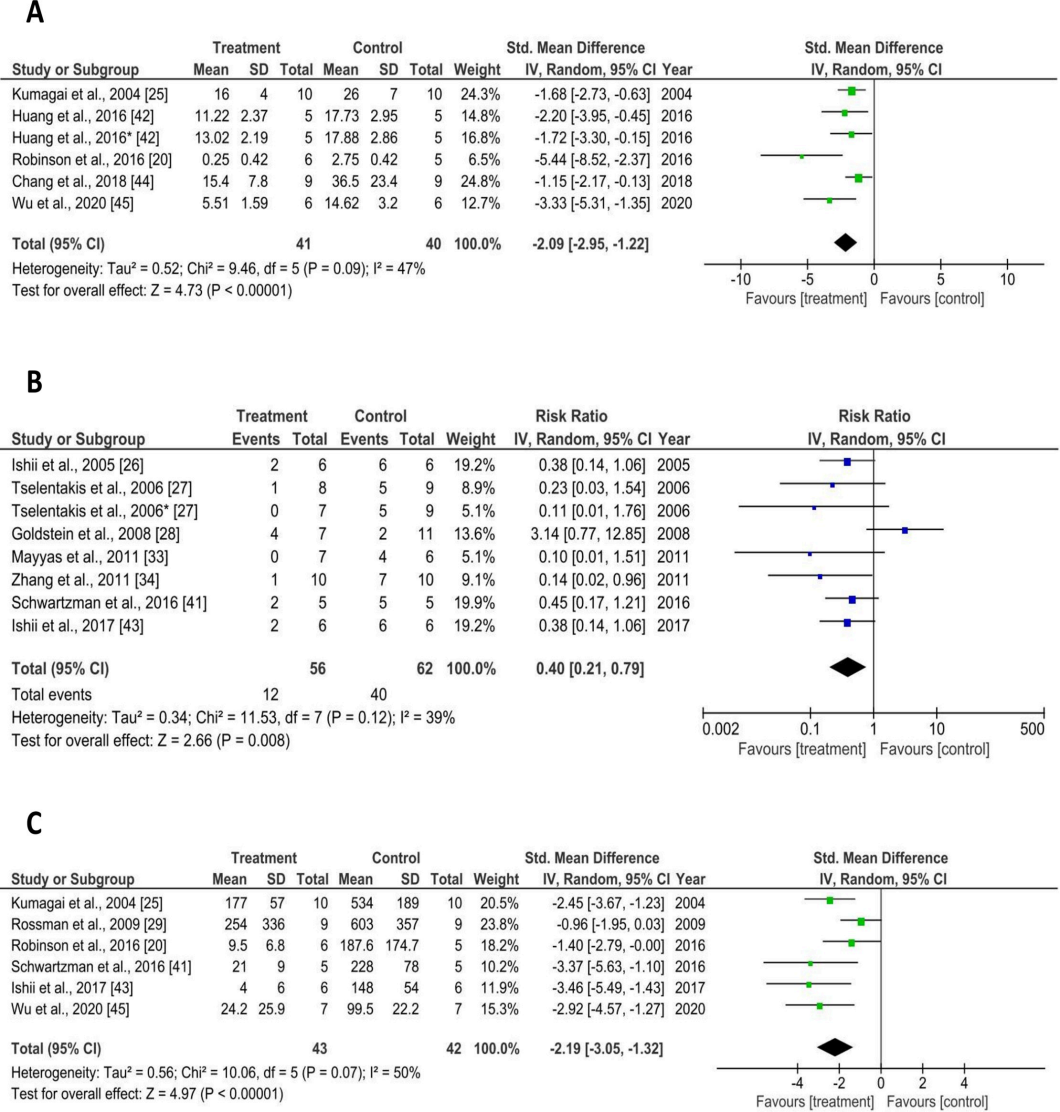
Forest plots of the effect of preclinical therapies on primary AF outcomes. (A) Standard mean difference of the degree of atrial fibrosis between POAF treatment and control groups following operation, (B) Risk ratio of AF inducibility in animals following operation, and (C) Standard mean difference of AF duration between POAF treatment and control groups following operation. CI = confidence interval; IV = inverse variance; SD = standard deviation.

To assess the current state of all pre-clinical therapies published to date, we performed a meta-analysis of their primary efficacy outcomes. As shown in Table 3 and Fig 2, grouped analysis showed that preclinical therapies altogether attenuated atrial fibrosis (SMD -2.09; 95% confidence interval [CI] -2.95 to -1.22; *p* < 0.00001; *I*^*2*^ = 47%), AF inducibility (RR 0.40; 95% CI 0.21 to 0.79; *p* = 0.008; *I*^*2*^ = 39%), and AF duration (SMD -2.19; 95% CI -3.05 to -1.32; *p* < 0.00001; *I*^*2*^ = 50%). Treatment with any preclinical therapy was also more apt to result in termination of AF.

**Table 3:** Differences in atrial fibrosis, AF duration, inducibility and termination.

		**Control**				**Treatment**			
**Reference**	**Error**	**Control**	**N**	**Mean**	**Error**	**Treatment**	**N**	**Mean**	**Error**
Atrial fibrosis*									
Kumagai et al., 2004 [25]	SD	SP control	10	26%	7%	Atorvastatin	10	16%	4%
Huang et al., 2016 [42]	SEM	SP control	5	17.73%	1.32%	S3I-201	5	11.22%	1.06%
Huang et al., 2016 [42]	SEM	SP control	5	17.88%	1.28%	miR-21	5	13.02%	0.98%
Robinson et al., 2016 [20]**	SD	PC control	5	2.75	0.42	PPX[AMIO,DEX]	6	0.25	0.42
Chang et al., 2018 [44]	SEM	opLA	9	36.5%	7.8%	MPT0E014	9	15.4%	2.6%
Wu et al., 2020 [45]	SEM	SP control	6	14.62%	1.31%	Colchicine	6	5.51%	0.65%
**AF duration**									
Kumagai et al., 2004 [25]	SD	SP control	10	534 s	189 s	Atorvastatin	10	177 s	57 s
Rossman et al., 2009 [29]	SEM	SP control	9	603 s	119 s	GAP-134	9	254 s	112 s
Schwartzman et al., 2016 [41]	SD	SP control	5	228 s	78 s	PBM	5	21 s	9 s
Robinson et al., 2016 [20]	SD	PC control	5	187.6 s	174.7 s	PPX[AMIO,DEX]	6	9.5 s	6.8 s
Ishii et al., 2017 [43]	SD	Atriotomy control	6	148 s	54 s	Methylprednisolone	6	4 s	6 s
Wu et al., 2020 [45]	SEM	SP control	7	99.5 s	8.4 s	Colchicine	7	24.2 s	9.8 s
		**Control**				**Treatment**			
		**Control**	**N total**	**N with event (%)**		**Treatment**	**N total**	**N with event (%)**	
**AF inducibility**									
Ishii et al., 2005 [26]		Atriotomy control	6	6 (100%)		Methylprednisolone	6	2 (33.3%)	
Tselentakis et al., 2006 [27]		SP control	9	5 (55.5%)		Methylprednisolone	8	1 (12.5%)	
Tselentakis et al., 2006 [27]		SP control	9	5 (55.5%)		Ibuprofen	7	0 (0%)	
Goldstein et al., 2008 [28] ^†^		SP control	11	2 (18.2%)		Prednisone	7	4 (57.1%)	
Mayyas et al.,2011 [33]		Atriotomy control	6	4 (66.6%)		n-3 PUFA	7	0 (0%)	
Zhang et al., 2011 [34]		SP control	10	7 (70%)		n-3 PUFA	10	1 (10%)	
Schwartzman et al., 2016 [41]		SP control	5	5 (100%)		Amiodarone	5	2 (40%)	
Ishii et al., 2017 [43]		Atriotomy control	6	6 (100%)		Methylprednisolone	6	2 (33.3%)	
**AF termination**									
Shimizu et al., 1993 [21]		-	-	-		E-4031	4	4 (100%)	
Goldstein et al., 2004 [24]		-	-	-		AZD7009	7	7 (100%)	
Matsumoto et al., 2010 [30]		-	-	-		Atorvastatin	11	10 (90.9%)	
Bhimani et al., 2014 [36]		-	-	-		Ranolazine	4	3 (75%)	
Sadrpour et al., 2015 [38]		-	-	-		M-II	2	2 (100%)	

*Masson’s trichrome was used for detection of collagen fibers in prepared atrial tissues. Results shown indicate mean % area fibrosis.

** Cardiac adhesion was assessed using a 4-point scoring system: 0 –no adhesions; 1 –mild adhesions; 2 –moderate adhesions; 3 –severe adhesions.

†only POD3 result shown.

For the purpose of meta-analysis, only the studies that reported (1) the percentage atrial fibrosis; (2) the number of animals in which AF was inducible; and (3) the mean duration of induced AF were reported. Studies were not included if they did not specify the type of arrhythmia (i.e., summed all atrial tachyarrhythmias). AF = atrial fibrillation; opLA = pericardiotomy of left atrium; PBM = Plasma-Based, Amiodarone-Impregnated material; PC = pericardiotomy; PPX[AMIO,DEX] = amiodarone- and dexamethasone-loaded parylene-C film; PUFA = polyunsaturated fatty acid; SD = standard deviation; SEM = standard error of the mean; SP = sterile pericarditis.

**Fig 3:**
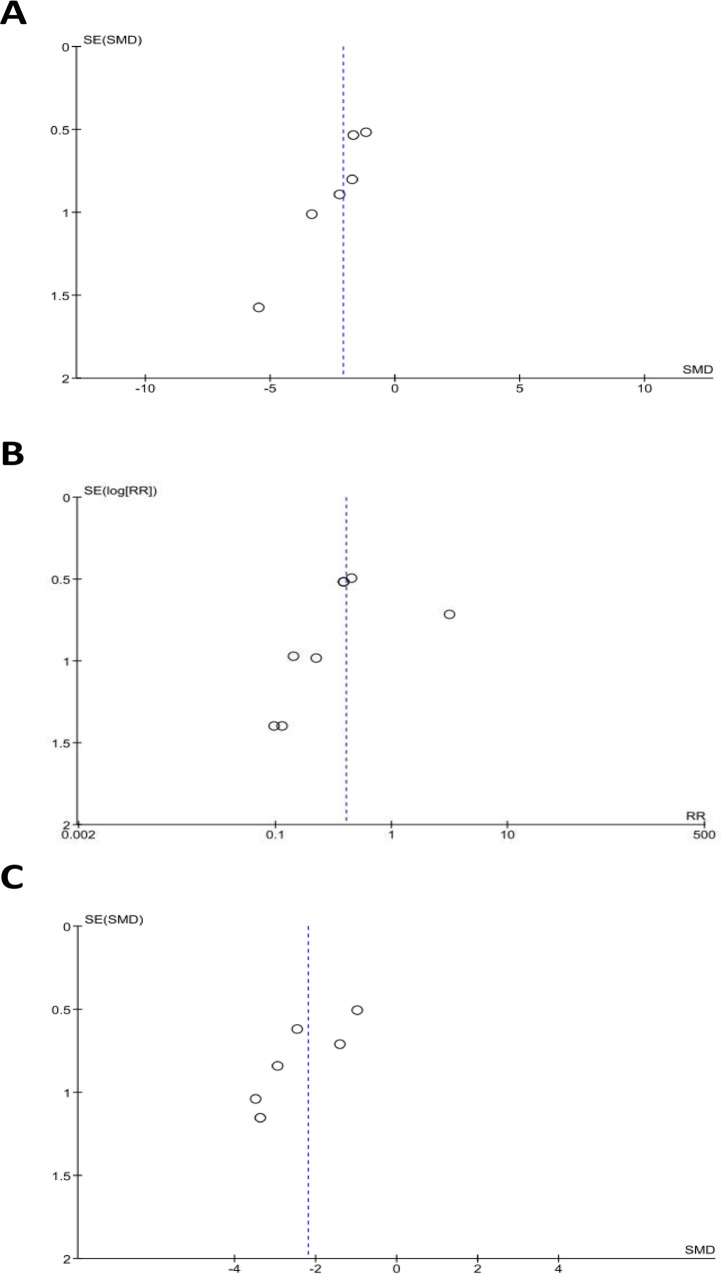
Funnel plot of publication bias in the meta-analysis of primary outcomes. (A) Atrial fibrosis, (B) AF inducibility, and (C) AF duration. RR = risk ratio; SE = standard error; SMD = standardized mean difference.

We performed a risk of bias assessment on all studies included in the present review using the SYRCLE bias tool (Table 4). Overall, the risk of bias was unclear for many; particularly with regards to selection (i.e., allocation concealment), performance (i.e., random housing, blinding of participants and personnel) and detection bias (i.e., random outcome assessment, blinding of outcome assessment). Certain interventions (e.g., methylprednisolone, vanoxerine) were only investigated by a single group so the generalizability of these findings needs to be confirmed and this impacted on the study quality which was assessed using the CAMARADES checklist (S3 Table in S1 File). Collectively, the median score was 4 (Interquartile Range [IQR] 3.75–5). While the majority of double-arm studies incorporated randomization protocol for assigning animals to treatment groups (11 of 18 studies), only one study assessed the dose-response relationship [45]. No study formally stated their sample size calculation and only four studies stated that investigators were blinded for assessment of outcomes. Although body temperature may influence cardiac electrophysiology [46], only 25% of studies stated physiologic temperatures were maintained during the procedures. Finally, we sought to assess any potential publication bias through a test for asymmetry using funnel plots. As shown in Fig 3, computed funnel plots for the three primary efficacy outcomes (i.e., AF fibrosis, AF inducibility, AF duration) illustrated a trend of asymmetry, with greater proportion of the outcomes favouring positive findings. This is potentially indicative of publication bias, however, given the small number of studies reporting each outcome measure, the plots must also be interpreted with caution as the power to detect bias is low.

**Table 4:** SYRCLE bias tool summary table.

● Low risk of bias	Selection bias			Performance bias		Detection bias		Attrition bias	Reporting bias	Other bias
● High risk of bias	Sequence generation	Baseline characteristics	Allocation concealment	Random housing	Blinding of participants and personnel	Random outcome assessment	Blinding of outcome assessment	Incomplete outcome data	Selective outcome reporting	Other bias
● Unclear										
○ Not applicable										
Reference										
**Double-arm Trials**										
Becker et al., 2002 [22]	●	●	●	●	●	●	●	●	●	●
Kumagai et al., 2004 [25]	●	●	●	●	●	●	●	●	●	●
Ishii et al., 2005 [26]	●	●	●	●	●	●	●	●	●	●
Tselentakis et al., 2006 [27]	●	●	●	●	●	●	●	●	●	●
Rossman et al., 2009 [29]	●	●	●	●	●	●	●	●	●	●
Yoo et al., 2010 [31]	●	●	●	●	●	●	●	●	●	●
Mayyas et al., 2011 [33]	●	●	●	●	●	●	●	●	●	●
Zhang et al., 2011 [34]	●	●	●	●	●	●	●	●	●	●
Schuessler et al., 2012 [35]	●	●	●	●	●	●	●	●	●	●
Fu et al., 2015 [37]	●	●	●	●	●	●	●	●	●	●
Zhang et al., 2015 [39]	●	●	●	●	●	●	●	●	●	●
Yang et al., 2015 [40]	●	●	●	●	●	●	●	●	●	●
Schwartzman et al., 2016 [41]	●	●	●	●	●	●	●	●	●	●
Huang et al., 2016 [42]	●	●	●	●	●	●	●	●	●	●
Robinson et al., 2016 [20]	●	●	●	●	●	●	●	●	●	●
Ishii et al., 2017 [43]	●	●	●	●	●	●	●	●	●	●
Chang et al., 2018 [44]	●	●	●	●	●	●	●	●	●	●
Wu et al., 2020 [45]	●	●	●	●	●	●	●	●	●	●
**Single-arm Trials**										
Shimizu et al., 1993 [21]	**○**	●	**○**	●	**○**	●	**○**	●	●	●
Kumagai et al., 2003 [23]	**○**	●	**○**	●	**○**	●	**○**	●	●	●
Goldstein et al., 2004 [24]	**○**	●	**○**	●	**○**	●	**○**	●	●	●
Goldstein et al., 2008 [28]	**○**	●	**○**	●	**○**	●	**○**	●	●	●
Matsumoto et al., 2010 [30]	**○**	●	**○**	●	**○**	●	**○**	●	●	●
Cakulev et al., 2011 [32]	**○**	●	**○**	●	**○**	●	**○**	●	●	●
Bhimani et al., 2014 [36]	**○**	●	**○**	●	**○**	●	**○**	●	●	●
Sadrpour et al., 2015 [38]	**○**	●	**○**	●	**○**	●	**○**	●	●	●

## Discussion

In this study, we report the published work to date exploring pre-clinical therapies for POAF. Amongst clinical AF syndromes, POAF is unique as it is highly prevalent, clinically meaningful, and demonstrates predictable time-course [1–5]. Given that standard therapies are not useful or highly toxic, these attributes combine to make the development of effective prophylaxis both clinically and commercially attractive. Although our review identified several promising pre-clinical strategies, clinical translation has been limited which may reflect the quality of the evidence, the impractical nature of the interventions or the risks of off target (systemic) complications.

The outcomes reported in this systematic review should be interpreted in light of several limitations. First, none of the animal models demonstrated spontaneous POAF. In all cases, atrial fibrillation was induced in animals via extra-stimulation (e.g., atrial burst pacing) following pericardiotomy, atriotomy, and/or application of sterile pericarditis. This artificial POAF-like state ignores the complex and multi-faceted electrophysiology that arises in human patients. Second, as shown in S4 Table in S1 File, induction protocols (i.e., pacing cycle length, pulse duration and voltage) and AF definitions varied considerably between studies which, in the absence of pre-registration, prompts speculation that these methods may have been customized to enhance outcomes. Furthermore, funnel plots, used to evaluate publication bias, showed a trend of asymmetry favouring successful interventions. Although it is challenging to definitively identify publication bias given the small number of studies (<10) that discourage the use of statistical tests for asymmetry [47, 48], the results presented in this review must be considered in light of these potential biases. It is also notable that the largest group studied in the entire sample comprised only 12 animals. This would be fine if the outcomes were extremely reproducible and justified by a robust sample size calculation, but no study published this important design feature. Finally, none of the models incorporated any of the risk factors for POAF that include advanced age, obesity, congestive heart failure, chronic renal failure, or lung disease [1, 2, 4]. All studies were performed in young healthy animals, or failed to report the age at experimentation. These shortcomings compromise external validity and reduce the ability of any preclinical model to be translated. Despite the complexity and cost needed to mimic human conditions, the results from this present study suggest that more clinically applicable animal models for POAF are desperately needed.

Despite the number of studies identified, we were discouraged to find that very few studies replicate key findings. When multiple studies used the same approach, they were often performed by the same group which limits generalizability of the findings. In fact, no study satisfied all the criteria needed to ensure low risk of bias as design issues were often not outlined. With the institution of consistent reporting standards across many peer reviewed journals, study quality and reproducibility will likely become more consistent and increase confidence in pre-clinical reports.

Progress in this field is also likely limited by reliance on large animal models (81% of the studies). The limited throughput and high cost of these large animal models help to explain the small group sizes and few treatments strategies used in these studies. Small animal models of POAF have only been developed in the past 4–5 years with 3 studies using a rat model of sterile pericarditis [37, 42, 45] and 2 study using a rabbit model of pericardiotomy [20, 44]. Small animal models open the possibility of broad compound screens and dose-response relationships for promising compounds prior to validation within large animal models. The latter still being necessary as these large animal models help confirm product efficacy and scaling to larger “human” doses. In the future, ex vivo heart preparations or pluripotent-derived cell models of POAF may emerge but this will likely be challenging as POAF arises from the complex interplay between surgical intervention, medical co-morbidities and an intact host.

The pathogenesis of POAF revolves about the interplay between inflammation, pre-existing triggers, structural disease, and peri-operative insults. As shown in S5 Table in S1 File, pre-clinical interventions focus on reducing inflammation, modifying cardiac electrophysiology or altering substrate with none addressing all 3 mechanisms. It is very possible that, for any therapy to provide a real-world meaningful reduction in POAF, a combinatorial approach will be needed. This insight may help to explain why clinical trials have failed to find a consistent signal for efficacy when a preclinical approach is applied to the clinic. For example, the pre-clinical data supporting pre-operative steroids to prevent POAF appears to be uniform with multiple studies showing enhanced AF termination, reduced AF inducibility, reduced atrial fibrosis and reduced conduction inhomogeneities. In contrast, the 16+ clinical trials to date investigating the ability of steroids to suppress POAF have failed to show a clear signal for benefit [49–53]. The interpretation of this aggregate data may have been complicated by drug selection, dose administered, trial-specific differences in logistics and even inadvertent toxicity (including corticosteroid-related AF). Peri-operative steroids have not been widely adopted or recommended in recent guidelines. Similar discrepancy in translation from preclinical animal studies to clinical trials is seen with prophylactic administration of PUFA [54], which demonstrated no effect on the incidence of POAF in patients undergoing open heart surgery despite strong evidence in animal models.

Future approaches for POAF will also likely evolve to include more biological therapies that target the fundamental substrate or triggers of POAF [55]. In this systematic review, only 5 studies explored the impact of biological intervention on POAF. These studies focused on predictable targets (inflammation and cardiac electrophysiology) but only recently have next generation targets (such as autonomic tone, atrial ectopy, mechano-electric feedback and hormonal modulation) become obvious [56].

The ideal therapy for POAF should be affordable, effective and non-toxic. When examined in this light, many of the approaches chosen to date fall short. The poor efficacy of antiarrhythmic drugs suggests that changing cellular electrophysiology alone is not likely to be effective but may provide benefit when combined with other strategies. Systemic immunomodulatory approaches are complicated by increased risks of infection, hyperglycemia, gastritis, pro-arrhythmia and myelosuppression. Anti-fibrotic approaches may be effective if confined to the atria but are unlikely to have much benefit if they impact post-operative healing or increase the risk of infection. Local delivery of a treatment to the atria avoids many of these systemic issues. This injectate has to persist long enough to modify cell function but, unlike a therapy for longstanding paroxysmal or persistent atrial fibrillation, a therapy for POAF need only be present during the post-operative period. A strategy that involves local injection of miRNA at the time of cardiac surgery to modify cellular electrophysiology or fibrosis for a few days would exemplify this approach. But the high price tag associated with “Good Manufacturing Practices” and xenogen free recombinant vectors scaled to human doses (100X greater than a rat) effectively preclude realistic consideration at this time. The challenge lies in identifying cost-effective solutions. Plausible options include: 1) modifying surgical techniques to limit epicardial contact (perhaps using artificial intelligence or robotic supported techniques) [57], 2) empiric substrate modification for high risk patients at the time of surgery, 3) epicardial application of biodegradable materials embedded with multiple proteins or transcripts that inhibit fibrosis and inflammation, or suppress early afterdepolarizations and delayed afterdepolarizations, 4) multimodal systemic therapies to decrease inflammation and pro-arrhythmia, or 5) pre-treating high risk patients using catheter-based modification of pro-arrhythmic substrate. As outlined, the emerging number of complimentary preclinical models will help identify promising therapies to treat this disease which is ripe for disruptive innovation.

## Supporting information

S1 Checklist PRISMA checklist. (DOC)

S1 File (DOCX)

S2 File PROSPERO systematic review protocol. (PDF)
