# Supplementary material for: Systematic review of pre-clinical therapies for post-operative atrial fibrillation
Source: PLoS One. 2020 Nov 4;15(11):e0241643. doi: 10.1371/journal.pone.0241643 (PMC7641461; doi:10.1371/journal.pone.0241643)
Supplement: S1 File — (DOCX) [file pone.0241643.s002.docx]

Table of Contents

S1 Table: Search strategy………………………………………………….………….…….….…2

S2 Table: Study design and outcomes……………………………………………..………….…29

S3 Table: CAMARADES checklist for study quality assessment……………………………....34

S4 Table: Anesthetic agents, AF induction, and AF definition used in animal studies…...…..…36

S5 Table: Comparison of primary outcome efficacy between intervention mechanisms…….…38

**S1 Table. Search strategy**

1. **Embase Classic+Embase** <1947 to 2020 September 03>

|  | **Search Strategy** |
| --- | --- |
| 1 | Exp animal experiment/ |
| 2 | Exp animal model/ |
| 3 | Exp invertebrate/ |
| 4 | Animal/ |
| 5 | Exp disease model/ |
| 6 | Chordata/ |
| 7 | Exp invertebrate Chordata/ |
| 8 | Vertebrate/ |
| 9 | Exp amphibia/ |
| 10 | Exp bird/ |
| 11 | Exp fish/ |
| 12 | Exp reptile/ |
| 13 | Mammal/ |
| 14 | Primate/ |
| 15 | Exp Artiodactyla/ |
| 16 | Exp Carnivora/ |
| 17 | Exp Cetacea/ |
| 18 | Exp bat/ |
| 19 | Exp elephant/ |
| 20 | Exp hyrax/ |
| 21 | Exp Insectivora/ |
| 22 | Exp lagomorph/ |
| 23 | Exp marsupial/ |
| 24 | Exp monotreme/ |
| 25 | Exp Perissodactyla/ |
| 26 | Exp rodent/ |
| 27 | Exp Scandentia/ |
| 28 | Exp Sirenia/ |
| 29 | Exp Xenarthra/ |
| 30 | Haplorhini/ |
| 31 | Exp prosimian/ |
| 32 | Exp Platyrrhini/ |
| 33 | Exp tarsiiform/ |
| 34 | Catarrhini/ |
| 35 | Exp Cercopithecidae/ |
| 36 | Exp Hylobatidae/ |
| 37 | Hominid/ |
| 38 | Exp Gorilla gorilla/ |
| 39 | Exp Pan paniscus/ |
| 40 | Exp Pan troglodytes/ |
| 41 | Exp Pongo pygmaeus/ |
| 42 | 1 or 2 or 3 or 4 or 5 or 6 or 7 or 8 or 9 or 10 or 11 or 12 or 13 or 14 or 15 or 16 or 17 or 18 or 19 or 20 or 21 or 22 or 23 or 24 or 25 or 26 or 27 or 28 or 29 or 30 or 31 or 32 or 33 or 34 or 35 or 36 or 37 or 38 or 39 or 40 or 41 |
| 43 | Animal*.tw. |
| 44 | Mice.tw. |
| 45 | Mus.tw. |
| 46 | Mouse.tw. |
| 47 | Murine.tw. |
| 48 | Woodmouse.tw. |
| 49 | Rat*.tw. |
| 50 | Murinae.tw. |
| 51 | Muridae.tw. |
| 52 | Cottonrat*.tw. |
| 53 | Hamster*.tw. |
| 54 | Cricetinae.tw. |
| 55 | Rodent*.tw. |
| 56 | Pig*.tw. |
| 57 | Swine*.tw. |
| 58 | Boar*.tw. |
| 59 | Sus scrofa.tw. |
| 60 | Ferret*.tw. |
| 61 | Polecat*.tw. |
| 62 | Mustela putorius.tw. |
| 63 | (guinea adj2 pig*).tw. |
| 64 | Cavia.tw. |
| 65 | Callithrix.tw. |
| 66 | Marmoset*.tw. |
| 67 | Cebuella.tw. |
| 68 | Hapale.tw. |
| 69 | Octodon.tw. |
| 70 | Chinchilla*.tw. |
| 71 | Gerbil*.tw. |
| 72 | Jird*.tw. |
| 73 | Merione*.tw. |
| 74 | Rabbit*.tw. |
| 75 | Hare*.tw. |
| 76 | Diptera.tw. |
| 77 | Flies.tw. |
| 78 | Fly.tw. |
| 79 | Dipteral.tw. |
| 80 | Drosophila.tw. |
| 81 | Drosophilidae.tw. |
| 82 | Cat*.tw. |
| 83 | Carus.tw. |
| 84 | Felis.tw. |
| 85 | Nematod*.tw. |
| 86 | Sipunculida.tw. |
| 87 | Dog*.tw. |
| 88 | Canine.tw. |
| 89 | Canis.tw. |
| 90 | Sheep*.tw. |
| 91 | Mouflon*.tw. |
| 92 | Ovis.tw. |
| 93 | Goat*.tw. |
| 94 | Capra*.tw. |
| 95 | Rupicapra.tw. |
| 96 | Chamois.tw. |
| 97 | Haplorhini.tw. |
| 98 | Monkey*.tw. |
| 99 | Anthropoidea.tw. |
| 100 | Anthropoids.tw. |
| 101 | Saguinus.tw. |
| 102 | Tamarin*.tw. |
| 103 | Leontopithecus.tw. |
| 104 | Hominidae.tw. |
| 105 | Ape*.tw. |
| 106 | Pan.tw. |
| 107 | Paniscus.tw. |
| 108 | (pan adj2 paniscus).tw. |
| 109 | Bonobo*.tw. |
| 110 | Troglodytes.tw. |
| 111 | (pan adj2 troglodytes).tw. |
| 112 | Gibbon*.tw. |
| 113 | Siamang*.tw. |
| 114 | Nomascus.tw. |
| 115 | Symphalangus.tw. |
| 116 | Chimpanzee*.tw. |
| 117 | (bush adj2 baby).tw. |
| 118 | (bush adj2 babies).tw. |
| 119 | Prosimian*.tw. |
| 120 | Galago*.tw. |
| 121 | Pongidae.tw. |
| 122 | Gorilla*.tw. |
| 123 | Pongo.tw. |
| 124 | Pygmaeus.tw. |
| 125 | (pongo adj2 pygmaeus).tw. |
| 126 | Orangutan*.tw. |
| 127 | Lemur*.tw. |
| 128 | Lemuridae.tw. |
| 129 | Horse*.tw. |
| 130 | Equus.tw. |
| 131 | Cow.tw. |
| 132 | Calf.tw. |
| 133 | Calves.tw. |
| 134 | Bull*.tw. |
| 135 | Chicken*.tw. |
| 136 | Gallus.tw. |
| 137 | Bird*.tw. |
| 138 | Quail.tw. |
| 139 | Poultr*.tw. |
| 140 | Fowl*.tw. |
| 141 | Reptile*.tw. |
| 142 | Reptilia.tw. |
| 143 | Snake*.tw. |
| 144 | Lizard*.tw. |
| 145 | Alligator*.tw. |
| 146 | Crocodile*.tw. |
| 147 | Turtle*.tw. |
| 148 | Amphibian*.tw. |
| 149 | Frog*.tw. |
| 150 | Bombina.tw. |
| 151 | Salientia.tw. |
| 152 | Toad*.tw. |
| 153 | (epidalea adj2 calamita).tw. |
| 154 | Salamander*.tw. |
| 155 | Eel*.tw. |
| 156 | Fish*.tw. |
| 157 | Pisces.tw. |
| 158 | Catfish*.tw. |
| 159 | Siluriformes.tw. |
| 160 | Arius.tw. |
| 161 | Heteropneustes.tw. |
| 162 | Sheatfish.tw. |
| 163 | Perch*.tw. |
| 164 | Percidae.tw. |
| 165 | Perca.tw. |
| 166 | Trout*.tw. |
| 167 | Char*.tw. |
| 168 | Salvelinus.tw. |
| 169 | (fathead adj2 minnow).tw. |
| 170 | Minnow.tw. |
| 171 | Cyprinidae.tw. |
| 172 | Carp.tw. |
| 173 | Zebrafish*.tw. |
| 174 | Goldfish*.tw. |
| 175 | Guppy.tw. |
| 176 | Guppies.tw. |
| 177 | Chub*.tw. |
| 178 | Tinca.tw. |
| 179 | Barbels.tw. |
| 180 | Barbus.tw. |
| 181 | Pimephales.tw. |
| 182 | Promelas.tw. |
| 183 | (poecilia adj2 reticulata).tw. |
| 184 | Mullet*.tw. |
| 185 | Seahorse*.tw. |
| 186 | (mugil adj2 curema).tw. |
| 187 | (atlantic adj2 cod).tw. |
| 188 | Shark*.tw. |
| 189 | Catshark.tw. |
| 190 | Anguilla.tw. |
| 191 | Salmonid*.tw. |
| 192 | Whitefish*.tw. |
| 193 | Salmon*.tw. |
| 194 | Sole.tw. |
| 195 | Solea.tw. |
| 196 | (sea adj2 lamprey).tw. |
| 197 | Lamprey*.tw. |
| 198 | Pumpkinseed.tw. |
| 199 | Sunfish*.tw. |
| 200 | Tilapia*.tw. |
| 201 | Turbot*.tw. |
| 202 | Flatfish*.tw. |
| 203 | Sciuridae.tw. |
| 204 | Squirrel*.tw. |
| 205 | Chipmunk*.tw. |
| 206 | Suslik*.tw. |
| 207 | Vole*.tw. |
| 208 | Lemming*.tw. |
| 209 | Muskrat*.tw. |
| 210 | Lemmus.tw. |
| 211 | Otter*.tw. |
| 212 | Marten*.tw. |
| 213 | Martes.tw. |
| 214 | Weasel.tw. |
| 215 | Badger*.tw. |
| 216 | Ermine.tw. |
| 217 | Mink*.tw. |
| 218 | Sable*.tw. |
| 219 | Gulo*.tw. |
| 220 | Wolverine*.tw. |
| 221 | Llama*.tw. |
| 222 | Mustela.tw. |
| 223 | Alpaca*.tw. |
| 224 | Camelid*.tw. |
| 225 | Guanaco*.tw. |
| 226 | Chiroptera*.tw. |
| 227 | Bat*.tw. |
| 228 | Fox*.tw. |
| 229 | Iguana*.tw. |
| 230 | (xenopus adj2 laevis).tw. |
| 231 | Parakeet*.tw. |
| 232 | Parrot*.tw. |
| 233 | Donkey*.tw. |
| 234 | Mule*.tw. |
| 235 | Zebra*.tw. |
| 236 | Shrew*.tw. |
| 237 | Bison*.tw. |
| 238 | Buffalo*.tw. |
| 239 | Deer*.tw. |
| 240 | Bear*.tw. |
| 241 | Panda*.tw. |
| 242 | (wild adj2 hog).tw. |
| 243 | (wild adj2 boar).tw. |
| 244 | Fitchew.tw. |
| 245 | Fitch.tw. |
| 246 | Beaver*.tw. |
| 247 | Jerboa*.tw. |
| 248 | Capybara*.tw. |
| 249 | 43 or 44 or 45 or 46 or 47 or 48 or 49 or 50 or 51 or 52 or 53 or 54 or 55 or 56 or 57 or 58 or 59 or 60 or 61 or 62 or 63 or 64 or 65 or 66 or 67 or 68 or 69 or 70 or 71 or 72 or 73 or 74 or 75 or 76 or 77 or 78 or 79 or 80 or 81 or 82 or 83 or 84 or 85 or 86 or 87 or 88 or 89 or 90 or 91 or 92 or 93 or 94 or 95 or 96 or 97 or 98 or 99 or 100 or 101 or 102 or 103 or 104 or 105 or 106 or 107 or 108 or 109 or 110 or 111 or 112 or 113 or 114 or 115 or 116 or 117 or 118 or 119 or 120 or 121 or 122 or 123 or 124 or 125 or 126 or 127 or 128 or 129 or 130 or 131 or 132 or 133 or 134 or 135 or 136 or 137 or 138 or 139 or 140 or 141 or 142 or 143 or 144 or 145 or 146 or 147 or 148 or 149 or 150 or 151 or 152 or 153 or 154 or 155 or 156 or 157 or 158 or 159 or 160 or 161 or 162 or 163 or 164 or 165 or 166 or 167 or 168 or 169 or 170 or 171 or 172 or 173 or 174 or 175 or 176 or 177 or 178 or 179 or 180 or 181 or 182 or 183 or 184 or 185 or 186 or 187 or 188 or 189 or 190 or 191 or 192 or 193 or 194 or 195 or 196 or 197 or 198 or 199 or 200 or 201 or 202 or 203 or 204 or 205 or 206 or 207 or 208 or 209 or 210 or 211 or 212 or 213 or 214 or 215 or 216 or 217 or 218 or 219 or 220 or 221 or 222 or 223 or 224 or 225 or 226 or 227 or 228 or 229 or 230 or 231 or 232 or 233 or 234 or 235 or 236 or 237 or 238 or 239 or 240 or 241 or 242 or 243 or 244 or 245 or 246 or 247 or 248 |
| 250 | Exp pericarditis/ |
| 251 | Exp heart surgery/ |
| 252 | Exp postoperative period/ |
| 253 | Exp heart atrium fibrillation/ or exp atrial fibrillation/ |
| 254 | Pericarditis.tw. |
| 255 | Cardiac surgical procedures.tw. |
| 256 | (cardiac adj2 surgery).tw. |
| 257 | (cardiac adj2 surgeries).tw. |
| 258 | (heart adj2 surgery).tw. |
| 259 | (heart adj2 surgeries).tw. |
| 260 | (post adj2 op).tw. |
| 261 | Post-op*.tw. |
| 262 | Postop.tw. |
| 263 | Postop*.tw. |
| 264 | (atrial adj2 fibrillation).tw. |
| 265 | Exp postoperative complication/ |
| 266 | Atriotomy.tw. |
| 267 | Pericardiotomy.tw. |
| 268 | 1 or 2 or 4 or 5 |
| 269 | 42 or 249 |
| 270 | 268 and 269 |
| 271 | 250 or 251 or 252 or 265 |
| 272 | 253 and 271 |
| 273 | 254 or 255 or 256 or 257 or 258 or 259 or 260 or 261 or 262 or 263 or 266 or 267 |
| 274 | 264 and 273 |
| 275 | 272 and 274 |
| 276 | 270 and 275 |

**2) Ovid MEDLINE(R) ALL** <1946 to September 3, 2020>

|  | **Search Strategy** |
| --- | --- |
| 1 | Exp Animal Experimentation/ |
| 2 | Exp Models, Animal/ |
| 3 | Exp Disease Models, Animal/ |
| 4 | Animals/ |
| 5 | Exp Invertebrates/ |
| 6 | Exp Animal Population Groups/ |
| 7 | Chordata/ |
| 8 | Exp Chordata, Nonvertebrate/ |
| 9 | Vertebrates/ |
| 10 | Exp Amphibians/ |
| 11 | Exp Birds/ |
| 12 | Exp Fishes/ |
| 13 | Exp Reptiles/ |
| 14 | Mammals/ |
| 15 | Primates/ |
| 16 | Exp Artiodactyla/ |
| 17 | Exp Carnivora/ |
| 18 | Exp Cetacea/ |
| 19 | Exp Chiroptera/ |
| 20 | Exp Elephants/ |
| 21 | Exp Hyraxes/ |
| 22 | Exp Insectivora/ |
| 23 | Exp Lagomorpha/ |
| 24 | Exp Marsupialia/ |
| 25 | Exp Monotremata/ |
| 26 | Exp Perissodactyla/ |
| 27 | Exp Rodentia/ |
| 28 | Exp Scandentia/ |
| 29 | Exp Sirenia/ |
| 30 | Exp Xenarthra/ |
| 31 | Haplorhini/ |
| 32 | Exp Strepsirhini/ |
| 33 | Exp Platyrrhini/ |
| 34 | Exp Tarsii/ |
| 35 | Catarrhini/ |
| 36 | Exp Cercopithecidae/ |
| 37 | Exp Hylobatidae/ |
| 38 | Hominidae/ |
| 39 | Exp Gorilla gorilla/ |
| 40 | Exp Pan paniscus/ |
| 41 | Exp Pan troglodytes/ |
| 42 | Exp Pongo pygmaeus/ |
| 43 | Animal*.tw. |
| 44 | Mice.tw. |
| 45 | Mus.tw. |
| 46 | Mouse.tw. |
| 47 | Murine.tw. |
| 48 | Woodmouse.tw. |
| 49 | Rat*.tw. |
| 50 | Murinae.tw. |
| 51 | Muridae.tw. |
| 52 | Cottonrat*.tw. |
| 53 | Hamster*.tw. |
| 54 | Cricetinae.tw. |
| 55 | Rodent*.tw. |
| 56 | Pig*.tw. |
| 57 | Swine*.tw. |
| 58 | Boar*.tw. |
| 59 | Sus scrofa.tw. |
| 60 | Ferret*.tw. |
| 61 | Polecat*.tw. |
| 62 | Mustela putorius.tw. |
| 63 | (guinea adj2 pig*).tw. |
| 64 | Cavia.tw. |
| 65 | Callithrix.tw. |
| 66 | Marmoset*.tw. |
| 67 | Cebuella.tw. |
| 68 | Hapale.tw. |
| 69 | Octodon.tw. |
| 70 | Chinchilla*.tw. |
| 71 | Gerbil*.tw. |
| 72 | Jird*.tw. |
| 73 | Merione*.tw. |
| 74 | Rabbit*.tw. |
| 75 | Hare*.tw. |
| 76 | Diptera.tw. |
| 77 | Flies.tw. |
| 78 | Fly.tw. |
| 79 | Dipteral.tw. |
| 80 | Drosophila.tw. |
| 81 | Drosophilidae.tw. |
| 82 | Cat*.tw. |
| 83 | Carus.tw. |
| 84 | Felis.tw. |
| 85 | Nematod*.tw. |
| 86 | Sipunculida.tw. |
| 87 | Dog*.tw. |
| 88 | Canine.tw. |
| 89 | Canis.tw. |
| 90 | Sheep*.tw. |
| 91 | Mouflon*.tw. |
| 92 | Ovis.tw. |
| 93 | Goat*.tw. |
| 94 | Capra*.tw. |
| 95 | Rupicapra.tw. |
| 96 | Chamois.tw. |
| 97 | Haplorhini.tw. |
| 98 | Monkey*.tw. |
| 99 | Anthropoidea.tw. |
| 100 | Anthropoids.tw. |
| 101 | Saguinus.tw. |
| 102 | Tamarin*.tw. |
| 103 | Leontopithecus.tw. |
| 104 | Hominidae.tw. |
| 105 | Ape*.tw. |
| 106 | Pan.tw. |
| 107 | Paniscus.tw. |
| 108 | (pan adj2 paniscus).tw. |
| 109 | Bonobo*.tw. |
| 110 | Troglodytes.tw. |
| 111 | (pan adj2 troglodytes).tw. |
| 112 | Gibbon*.tw. |
| 113 | Siamang*.tw. |
| 114 | Nomascus.tw. |
| 115 | Symphalangus.tw. |
| 116 | Chimpanzee*.tw. |
| 117 | (bush adj2 baby).tw. |
| 118 | (bush adj2 babies).tw. |
| 119 | Prosimian*.tw. |
| 120 | Galago*.tw. |
| 121 | Pongidae.tw. |
| 122 | Gorilla*.tw. |
| 123 | Pongo.tw. |
| 124 | Pygmaeus.tw. |
| 125 | (pongo adj2 pygmaeus).tw. |
| 126 | Orangutan*.tw. |
| 127 | Lemur*.tw. |
| 128 | Lemuridae.tw. |
| 129 | Horse*.tw. |
| 130 | Equus.tw. |
| 131 | Cow.tw. |
| 132 | Calf.tw. |
| 133 | Calves.tw. |
| 134 | Bull*.tw. |
| 135 | Chicken*.tw. |
| 136 | Gallus.tw. |
| 137 | Bird*.tw. |
| 138 | Quail.tw. |
| 139 | Poultr*.tw. |
| 140 | Fowl*.tw. |
| 141 | Reptile*.tw. |
| 142 | Reptilia.tw. |
| 143 | Snake*.tw. |
| 144 | Lizard*.tw. |
| 145 | Alligator*.tw. |
| 146 | Crocodile*.tw. |
| 147 | Turtle*.tw. |
| 148 | Amphibian*.tw. |
| 149 | Amphibia.tw. |
| 150 | Frog*.tw. |
| 151 | Bombina.tw. |
| 152 | Salientia.tw. |
| 153 | Toad*.tw. |
| 154 | (epidalea adj2 calamita).tw. |
| 155 | Salamander*.tw. |
| 156 | Eel*.tw. |
| 157 | Fish*.tw. |
| 158 | Pisces.tw. |
| 159 | Catfish*.tw. |
| 160 | Siluriformes.tw. |
| 161 | Arius.tw. |
| 162 | Heteropneustes.tw. |
| 163 | Sheatfish.tw. |
| 164 | Perch*.tw. |
| 165 | Percidae.tw. |
| 166 | Perca.tw. |
| 167 | Trout*.tw. |
| 168 | Char*.tw. |
| 169 | Salvelinus.tw. |
| 170 | (fathead adj2 minnow).tw. |
| 171 | Minnow.tw. |
| 172 | Cyprinidae.tw. |
| 173 | Carp.tw. |
| 174 | Zebrafish*.tw. |
| 175 | Goldfish*.tw. |
| 176 | Guppy.tw. |
| 177 | Guppies.tw. |
| 178 | Chub*.tw. |
| 179 | Tinca.tw. |
| 180 | Barbels.tw. |
| 181 | Barbus.tw. |
| 182 | Pimephales.tw. |
| 183 | Promelas.tw. |
| 184 | (poecilia adj2 reticulata).tw. |
| 185 | Mullet*.tw. |
| 186 | Seahorse*.tw. |
| 187 | (mugil adj2 curema).tw. |
| 188 | (atlantic adj2 cod).tw. |
| 189 | Shark*.tw. |
| 190 | Catshark.tw. |
| 191 | Anguilla.tw. |
| 192 | Salmonid*.tw. |
| 193 | Whitefish*.tw. |
| 194 | Salmon*.tw. |
| 195 | Sole.tw. |
| 196 | Solea.tw. |
| 197 | (sea adj2 lamprey).tw. |
| 198 | Lamprey*.tw. |
| 199 | Pumpkinseed.tw. |
| 200 | Sunfish*.tw. |
| 201 | Tilapia*.tw. |
| 202 | Turbot*.tw. |
| 203 | Flatfish*.tw. |
| 204 | Sciuridae.tw. |
| 205 | Squirrel*.tw. |
| 206 | Chipmunk*.tw. |
| 207 | Suslik*.tw. |
| 208 | Vole*.tw. |
| 209 | Lemming*.tw. |
| 210 | Muskrat*.tw. |
| 211 | Lemmus.tw. |
| 212 | Otter*.tw. |
| 213 | Marten*.tw. |
| 214 | Martes.tw. |
| 215 | Weasel.tw. |
| 216 | Badger*.tw. |
| 217 | Ermine.tw. |
| 218 | Mink*.tw. |
| 219 | Sable*.tw. |
| 220 | Gulo*.tw. |
| 221 | Wolverine*.tw. |
| 222 | Llama*.tw. |
| 223 | Mustela.tw. |
| 224 | Alpaca*.tw. |
| 225 | Camelid*.tw. |
| 226 | Guanaco*.tw. |
| 227 | Chiroptera*.tw. |
| 228 | Bat*.tw. |
| 229 | Fox*.tw. |
| 230 | Iguana*.tw. |
| 231 | (xenopus adj2 laevis).tw. |
| 232 | Parakeet*.tw. |
| 233 | Parrot*.tw. |
| 234 | Donkey*.tw. |
| 235 | Mule*.tw. |
| 236 | Zebra*.tw. |
| 237 | Shrew*.tw. |
| 238 | Bison*.tw. |
| 239 | Buffalo*.tw. |
| 240 | Deer*.tw. |
| 241 | Bear*.tw. |
| 242 | Panda*.tw. |
| 243 | (wild adj2 hog).tw. |
| 244 | (wild adj2 boar).tw. |
| 245 | Fitchew.tw. |
| 246 | Fitch.tw. |
| 247 | Beaver*.tw. |
| 248 | Jerboa*.tw. |
| 249 | Capybara*.tw. |
| 250 | Exp Pericarditis/ |
| 251 | Exp Cardiac Surgical Procedures/ |
| 252 | Exp Postoperative Period/ |
| 253 | Exp Postoperative Complications/ |
| 254 | Exp Atrial Fibrillation/ |
| 255 | Pericarditis.tw. |
| 256 | Cardiac Surgical Procedures.tw. |
| 257 | (Cardiac adj2 Surgery).tw. |
| 258 | (Cardiac adj2 Surgeries).tw. |
| 259 | (Heart adj2 Surgery).tw. |
| 260 | (Heart adj2 Surgeries).tw. |
| 261 | Atriotomy.tw. |
| 262 | Pericardiotomy.tw. |
| 263 | (Post adj2 op).tw. |
| 264 | Post-op*.tw. |
| 265 | Postop*.tw. |
| 266 | (Atrial adj2 Fibrillation).tw. |
| 267 | 1 or 2 or 3 or 4 |
| 268 | 43 or 44 or 45 or 46 or 47 or 48 or 49 or 50 or 51 or 52 or 53 or 54 or 55 or 56 or 57 or 58 or 59 or 60 or 61 or 62 or 63 or 64 or 65 or 66 or 67 or 68 or 69 or 70 or 71 or 72 or 73 or 74 or 75 or 76 or 77 or 78 or 79 or 80 or 81 or 82 or 83 or 84 or 85 or 86 or 87 or 88 or 89 or 90 or 91 or 92 or 93 or 94 or 95 or 96 or 97 or 98 or 99 or 100 or 101 or 102 or 103 or 104 or 105 or 106 or 107 or 108 or 109 or 110 or 111 or 112 or 113 or 114 or 115 or 116 or 117 or 118 or 119 or 120 or 121 or 122 or 123 or 124 or 125 or 126 or 127 or 128 or 129 or 130 or 131 or 132 or 133 or 134 or 135 or 136 or 137 or 138 or 139 or 140 or 141 or 142 or 143 or 144 or 145 or 146 or 147 or 148 or 149 or 150 or 151 or 152 or 153 or 154 or 155 or 156 or 157 or 158 or 159 or 160 or 161 or 162 or 163 or 164 or 165 or 166 or 167 or 168 or 169 or 170 or 171 or 172 or 173 or 174 or 175 or 176 or 177 or 178 or 179 or 180 or 181 or 182 or 183 or 184 or 185 or 186 or 187 or 188 or 189 or 190 or 191 or 192 or 193 or 194 or 195 or 196 or 197 or 198 or 199 or 200 or 201 or 202 or 203 or 204 or 205 or 206 or 207 or 208 or 209 or 210 or 211 or 212 or 213 or 214 or 215 or 216 or 217 or 218 or 219 or 220 or 221 or 222 or 223 or 224 or 225 or 226 or 227 or 228 or 229 or 230 or 231 or 232 or 233 or 234 or 235 or 236 or 237 or 238 or 239 or 240 or 241 or 242 or 243 or 244 or 245 or 246 or 247 or 248 or 249 |
| 269 | 1 or 2 or 3 or 4 or 5 or 6 or 7 or 8 or 9 or 10 or 11 or 12 or 13 or 14 or 15 or 16 or 17 or 18 or 19 or 20 or 21 or 22 or 23 or 24 or 25 or 26 or 27 or 28 or 29 or 30 or 31 or 32 or 33 or 34 or 35 or 36 or 37 or 38 or 39 or 40 or 41 or 42 |
| 270 | 268 or 269 |
| 271 | 267 and 270 |
| 272 | 250 or 251 or 252 or 253 |
| 273 | 254 and 272 |
| 274 | 255 or 256 or 257 or 258 or 259 or 260 or 261 or 262 or 263 or 264 or 265 |
| 275 | 266 and 274 |
| 276 | 273 or 275 |
| 277 | 271 and 276 |

**S2 Table. Study design and outcomes.**

| **Reference** | **Study Design** | **Primary outcome** | **Study Endpoint** | **Animals (treatment / control)** | **Secondary EP outcomes** | **Secondary structural outcomes** | **Secondary molecular outcomes** |
| --- | --- | --- | --- | --- | --- | --- | --- |
| Shimizu et al., 1993^1^ | Single arm | AF inducibility | 3 days | 12/- | AERP, CL, CT | - | - |
| Becker et al., 2002^2^ | Single arm | AF inducibility | 4-6 days | 12/- | CL | - | - |
| Kumagai et al., 2003^3^ | Single arm | AF inducibility | 2 days | 9/- | AERP, CT, CL, ECG | - | - |
| Goldstein et al., 2004^4^ | Single arm | AF termination & inducibility | 3-4 days | 11/- | capture threshold, AERP, CT, CL, activation mapping, ECG | - | - |
| Kumagai et al., 2004^5^ | Double arm | AF inducibility & atrial fibrosis | 2 days | 10/10 | ECG, AERP, CT | Atrial inflammation | CRP |
| Ishii et al., 2005^6^ | Double arm | Atrial conduction pattern | 3 days | 6/6 | AERP, activation mapping | Atrial inflammation | - |
| Tselentakis et al., 2006^7^ | Double arm | AF inducibility | 3 days | 7/9^†^  8/9^‡^ | CV, CL, RP, activation mapping | - | - |
| Goldstein et al., 2008^8^ | Double arm | AF inducibility | 4 days | 11/12 | CL, capture threshold, AERP, activation mapping | Atrial inflammation | CRP |
| Rossman et al., 2009^9^ | Double arm | AF inducibility | 2-3 days | 9/7 | CT, AERP, CV | - | Cx43 |
| Matsumoto et al., 2010^10^ | Single arm | AF termination | 3-4 days | 9/- | ECG, CL, capture threshold, AERP, CT | - | - |
| Yoo et al., 2010^11^ | Double arm | AF inducibility | 5 or 6 days | 9/6 | AERP | Atrial inflammation | - |
| Cakulev et al., 2011^12^ | Single arm | AF termination & inducibility | 4 days | 5/- | AERP, CT, CL, capture threshold, ECG | - | - |
| Mayyas et al., 2011^13^ | Double arm | Atrial inflammation | 2 or 4 days | 9/11 | HR, HRV, AERP | IHC | CRP, ET-1, Cx43, iNOS, IP3R-I |
| Zhang et al., 2011^14^ | Double arm | AF inducibility | 2 days | 10/10 | ECG, AERP, CT | Atrial inflammation | CRP, TNF-α, IL-6 |
| Schuessler et al., 2012^15^ | Double arm | HR & HRV | 3 days | 6/6 | PAD, SNRT, CT | Atrial inflammation | CRP |
| Bhimani et al., 2014^16^ | Single arm | AF termination | 3 or 4 days | 6/- | AERP, CT, CL, activation mapping | - | - |
| Fu et al., 2015^17^ | Double arm | AF inducibility & atrial fibrosis | 4 days | NR | ECG, AERP, AVNRP | - | MMPs, TIMPs, Col-1, Col-3, α-SMA, IL-1β, IL-6, TGF-β, IL-17A |
| Sadrpour et al., 2015^18^ | Single arm | AF termination | 3-4 days | 6/- | capture threshold, CL, CT, AERP, ECG, activation mapping | - | - |
| Zhang et al., 2015^19^ | Double arm | AF inducibility & atrial fibrosis | 21 days | 5/5 | AERP, CV | - | CRP, TNF-α, IL-6 |
| Yang et al., 2015^20^ | Double arm | AF inducibility  & atrial fibrosis | 56 days | 6/6 | activation mapping, CT | - | - |
| Schwartzman et al., 2016^21^ | Double arm | AF inducibility | 7 days | 5/5 | AERP, CT | Atrial inflammation | - |
| Huang et al., 2016^22^ | Double arm | Atrial fibrosis | 3 days | 5/5 | ECG, activation mapping | IHC | Col-1, Col-3, α-SMA, TNF-α, IL-1β, IL-6, TGF-β, STAT3, miR-21 |
| Robinson et al., 2016^23^ | Double arm | AF duration & atrial fibrosis | 4 weeks | 6/5 | - | Atrial inflammation | IL-6 |
| Ishii et al., 2017^24^ | Double arm | Atrial conduction pattern | 3 days | 6/6 | activation mapping | Atrial inflammation | - |
| Chang et al., 2018^25^ | Double arm | AF inducibility & atrial fibrosis | 7 days | 8/11 | APC, RMP, APA, APD | - | - |
| Wu et al., 2020^26^ | Double arm | AF duration & atrial fibrosis | 3 days | 7/7 | activation mapping, threshold stimulus, Wenckebach periodicity, SNRT, AVNRP | Atrial inflammation | IL-1β, IL-6, α-SMA, Col-1, Col-3, STAT3, p-AKT, p-P38, P38, JNK, p-ERK, ERK, p-P65, P65 |

^†^ Ibuprofen group vs. control

^‡^ Methylprednisolone group vs. control

APA = action potential amplitude; APC = atrial premature contractions; APD = action potential duration; AERP = atrial effective refractory period; AT = atrial tachyarrhythmia; CL = cycle length; CRP = C-reactive protein; CT = conduction time; CV = conduction velocity; EP = electrophysiological; HR = heart rate; HRV = heart rate variability; IHC = immunohistochemistry; iNOS = inducible nitric oxide synthase; NR = not reported; PAD = premature atrial depolarization, RMP = resting membrane potential; RP = refractory period; SNRT = sinus node recovery time

**S3 Table. CAMARADES checklist for study quality assessment.**

| **●** Fulfilling the criterion  **●** Not fulfilling the criterion  **●** Unclear  **○** Not applicable  **Reference** | **Peer reviewed publication** | **Randomization of subjects** | **Assessment of dose-response relationship** | **Statement of control of temperature** | **Sample size calculation** | **Blinded assessment of outcome** | **Compliance with animal welfare regulations** | **Statement of potential conflict of interest** | **Use of suitable animal model** | **Quality score (out of 9)** |
| --- | --- | --- | --- | --- | --- | --- | --- | --- | --- | --- |
| Shimizu et al., 1993^1^ | **●** | **○** | **●** | **●** | **●** | **○** | **●** | **●** | **●** | 4 |
| Becker et al., 2002^2^ | **●** | **●** | **●** | **●** | **●** | **●** | **●** | **●** | **●** | 5 |
| Kumagai et al., 2003^3^ | **●** | **○** | **●** | **●** | **●** | **○** | **●** | **●** | **●** | 3 |
| Goldstein et al., 2004^4^ | **●** | **○** | **●** | **●** | **●** | **○** | **●** | **●** | **●** | 3 |
| Kumagai et al., 2004^5^ | **●** | **●** | **●** | **●** | **●** | **●** | **●** | **●** | **●** | 5 |
| Ishii et al., 2005^6^ | **●** | **●** | **●** | **●** | **●** | **●** | **●** | **●** | **●** | 4 |
| Tselentakis et al., 2006^7^ | **●** | **●** | **●** | **●** | **●** | **●** | **●** | **●** | **●** | 3 |
| Goldstein et al., 2008^8^ | **●** | **○** | **●** | **●** | **●** | **○** | **●** | **●** | **●** | 4 |
| Rossman et al., 2009^9^ | **●** | **●** | **●** | **●** | **●** | **●** | **●** | **●** | **●** | 3 |
| Matsumoto et al., 2010^10^ | **●** | **○** | **●** | **●** | **●** | **○** | **●** | **●** | **●** | 4 |
| Yoo et al., 2010^11^ | **●** | **●** | **●** | **●** | **●** | **●** | **●** | **●** | **●** | 5 |
| Cakulev et al., 2011^12^ | **●** | **○** | **●** | **●** | **●** | **○** | **●** | **●** | **●** | 4 |
| Mayyas et al., 2011^13^ | **●** | **●** | **●** | **●** | **●** | **●** | **●** | **●** | **●** | 5 |
| Zhang et al., 2011^14^ | **●** | **●** | **●** | **●** | **●** | **●** | **●** | **●** | **●** | 5 |
| Schuessler et al., 2012^15^ | **●** | **●** | **●** | **●** | **●** | **●** | **●** | **●** | **●** | 4 |
| Bhimani et al., 2014^16^ | **●** | **○** | **●** | **●** | **●** | **○** | **●** | **●** | **●** | 5 |
| Fu et al., 2015^17^ | **●** | **●** | **●** | **●** | **●** | **●** | **●** | **●** | **●** | 3 |
| Sadrpour et al., 2015^18^ | **●** | **○** | **●** | **●** | **●** | **○** | **●** | **●** | **●** | 4 |
| Zhang et al., 2015^19^ | **●** | **●** | **●** | **●** | **●** | **●** | **●** | **●** | **●** | 5 |
| Yang et al., 2015^20^ | **●** | **●** | **●** | **●** | **●** | **●** | **●** | **●** | **●** | 3 |
| Schwartzman et al., 2016^21^ | **●** | **●** | **●** | **●** | **●** | **●** | **●** | **●** | **●** | 6 |
| Huang et al., 2016^22^ | **●** | **●** | **●** | **●** | **●** | **●** | **●** | **●** | **●** | 4 |
| Robinson et al., 2016^23^ | **●** | **●** | **●** | **●** | **●** | **●** | **●** | **●** | **●** | 5 |
| Ishii et al., 2017^24^ | **●** | **●** | **●** | **●** | **●** | **●** | **●** | **●** | **●** | 5 |
| Chang et al., 2018^25^ | **●** | **●** | **●** | **●** | **●** | **●** | **●** | **●** | **●** | 6 |
| Wu et al., 2020^26^ | **●** | **●** | **●** | **●** | **●** | **●** | **●** | **●** | **●** | 5 |

**S4 Table. Anesthetic agents, AF induction, and AF definition used in animal studies.**

| **Reference** | **Anesthesia** | **AF induction** | **AF definition** |
| --- | --- | --- | --- |
| Shimizu et al., 1993^1^ | IV pentobarbital (20-30 mg/kg) | Introduction of one premature atrial beat after a train of eight paced beats at cycle lengths of 400, 300, 200, or 150 ms; or rapid atrial pacing for 1-10 s | Rapid atrial rhythm (rate > 260 beats/min) characterized by variability of the beat-to-beat cycle length, polarity, morphology and amplitude of recorded bipolar electrograms |
| Becker et al., 2002^2^ | IV pentobarbital (0.5 mg/kg), maintained with halothane | Asynchronous pacing at a cycle length of 1000 ms at four times diastolic threshold from randomly selected right or left atrial pacing sites during sinus rhythm | Rapid atrial rhythm (rate > 260 beats/min) characterized by variability of the beat-to-beat cycle length, polarity, morphology and amplitude of recorded bipolar electrograms |
| Kumagai et al., 2003^3^ | IV pentobarbital (25 mg/kg), maintained with halothane | Burst atrial pacing of 90 to 110 ms for 20 beats | Rapid atrial rhythm with variability of the beat-to-beat cycle length, polarity, morphology and amplitude of recorded bipolar electrograms |
| Goldstein et al., 2004^4^ | IV pentothal (15 mg/kg), maintained with halothane | Burst atrial pacing or programmed stimulation | NS |
| Kumagai et al., 2004^5^ | IV pentobarbital (25 mg/kg), maintained with halothane | Burst atrial pacing of 90 to 110 ms for 20 beats | Rapid atrial rhythm with variability of the beat-to-beat cycle length, polarity, morphology and amplitude of recorded bipolar electrograms |
| Ishii et al., 2005^6^ | IV propofol (5-7 mg/kg), maintained with isoflurane | Burst atrial pacing at cycle lengths from 90 to 10 ms decremented by 10 ms | Average atrial activation interval <150 ms, with irregular electrogram morphology and rhythm |
| Tselentakis et al., 2006^7^ | NS xylazine (dose not specified), maintained with isoflurane | Burst pacing for 10 s at cycle lengths of 60, 70, and 80 ms | NS |
| Goldstein et al., 2008^8^ | IV pentothal (15 mg/kg), maintained with halothane | Programmed stimulation using up to three atrial premature beats following an eight-beat drive train at 200 ms; and rapid, burst pacing at cycle lengths down to 60 ms | NS |
| Rossman et al., 2009^9^ | IV pentobarbital (30 mg/kg), maintained with halothane | Rapid atrial pacing, using twice-threshold stimuli (pulse duration, 1.8 ms; 5 s train duration) starting at 500 bpm and increasing at increments of 20 bpm until 800 bpm | NS |
| Matsumoto et al., 2010^10^ | IV pentothal (15 mg/kg), maintained with halothane | Rapid atrial pacing for ≥20 beats performed from each of the atrial electrode sites beginning at a rate of 500 bpm with the rate incremented by 20 bpm until a rate of 800 bpm was reached or AF was achieved. | Rapid atrial rhythm with variability of the beat-to-beat cycle length, polarity, morphology and amplitude of recorded bipolar electrograms |
| Yoo et al., 2010^11^ | IV thiopental (10 mg/kg), maintained with isoflurane | Burst atrial pacing for 30 s starting at a cycle length of 450 ms, decrementing by 10 ms until 100 ms | Irregular tachycardia above 300 bpm and any tachycardias at rates above 350 bpm |
| Cakulev et al., 2011^12^ | IV pentothal (15 mg/kg), maintained with halothane | Programmed stimulation using up to three atrial premature beats following an eight-beat drive train at 200 ms; and rapid, burst pacing at cycle lengths down to 60 ms | Rapid atrial rhythm with variability of the beat-to-beat cycle length, polarity, morphology and amplitude of recorded bipolar electrograms |
| Mayyas et al., 2011^13^ | Not stated | Burst atrial pacing | NS |
| Zhang et al., 2011^14^ | IM pentobarbital (30 mg/kg), maintained with isoflurane | Burst atrial pacing using 10 V, 10 ms pulse duration at 10 Hz for 10 s | Rapid irregular atrial arrhythmia with mean cycle length <150 ms and lasting > 1 min |
| Schuessler et al., 2012^15^ | IV propofol (5-7 mg/kg), maintained with isoflurane | N/A | N/A |
| Bhimani et al., 2014^16^ | IV pentothal (15 mg/kg), maintained with halothane for SP induction; IV pentobarbital (30 mg/kg), maintained with isoflurane for open-chest study | Rapid atrial pacing for ≥20 beats performed from each of the atrial electrode sites beginning at a rate of 500 bpm with the rate incremented by 20 bpm until a rate of 800 bpm was reached or AF was achieved. | NS |
| Fu et al., 2015^17^ | NS pentobarbital (40 mg/kg) | 5 consecutive bursts of rapid stimulation (25, 30, 40, 50 and 83 Hz) for 30 sec | Rapid and fragmented atrial electrograms with an irregular ventricular rhythm for at least 1 sec immediately following the burst pacing |
| Sadrpour et al., 2015^18^ | IV pentothal (15 mg/kg), maintained with halothane | Programmed stimulation using up to three atrial premature beats following an eight-beat drive train at 200 ms; and rapid, burst pacing at cycle lengths down to 60 ms | NS |
| Zhang et al., 2015^19^ | IV pentobarbital (30 mg/kg) | NS | NS |
| Yang et al., 2015^20^ | IV propofol (2 mg/kg) | Continuous S1 and programmed S1S2 stimuli at the RAA with decremental pacing down to the atrial refractory period (+isoproterenol) | Fast, irregular atrial activation (>300 bpm) |
| Schwartzman et al., 2016^21^ | Inhaled isoflurane | 30 sec burst atrial pacing for 150 ms | Varying atrial morphology and irregularly irregular ventricular response on surface electrocardiogram |
| Huang et al., 2016^22^ | IP pentobarbital (40 mg/kg) | 5 consecutive 30 sec burst atrial pacing (25, 30, 40, 50, and 83 Hz) at 3 min intervals | Rapid and fragmented atrial electrograms with irregular atrioventricular nodal conduction and ventricular rhythm for at least 2 seconds |
| Robinson et al., 2016^23^ | Ketamine (40 mg/kg) and xylazine (7 mg/kg) maintained with 1.5-2.5% isoflurane | Exposure to an International Electrotechnical Commission 6LR61 alkaline battery | High-frequency, low-amplitude atrial electrograms, typically with varying R-R intervals |
| Ishii et al., 2017^24^ | IV propofol (5-7 mg/kg), maintained with isoflurane | Burst pacing of the left atrium at cycle lengths from 90 to 10 ms decremented by 10 ms | Atrial activation interval of <150 ms, with an irregular electrogram morphology and rate |
| Chang et al., 2018^25^ | Inhaled isoflurane (5%) and IP pentobarbital | Rapid atrial pacing at 20 Hz for 1 sec | NS |
| Wu et al., 2020^26^ | IP sodium pentobarbital (40 mg/kg) | 5 consecutive bursts of rapid stimulation (25, 30, 40, 60, 83 Hz) for 30 s at 3-min interval | Period of rapid irregular atrial rhythm lasting >5 s |

IM = intramuscular; IP = intraperitoneal; IV = intravenous; NS = not specified.

**S5 Table. Comparison of primary outcome efficacy between intervention mechanisms.**

| ● Demonstrated efficacy | | | ● Unclear | | |
| --- | --- | --- | --- | --- | --- |
| ● Demonstrated opposite effect | | | ○ Demonstrated no effect | | |
|  | **Primary outcome efficacy** | | | | |
| **References** | **Atrial fibrosis†** | **AF inducibility** | | **AF duration** | **AF termination** |
| **Reduction of inflammation** | | | | | |
| Kumagai et al., 2004^5^ | ● |  | | ● |  |
| Ishii et al., 2005^6^ |  | ● | | ● |  |
| Tselentakis et al., 2006^7^ | ● | ● | |  |  |
| Goldstein et al., 2008^8^ | ● | ● | |  |  |
| Yoo et al., 2010^11^ | ● | ○ | | ○ |  |
| Mayyas et al., 2011^13^ |  | ● | |  |  |
| Zhang et al., 2011^14^ |  | ● | | ● |  |
| Schuessler et al., 2012^15^ |  |  | |  |  |
| Fu et al., 2015^17^ | ● | ● | | ● |  |
| Zhang et al., 2015^19^ | ● | ○ | | ● |  |
| Huang et al., 2016^22^ | ● | ● | | ● |  |
| Ishii et al., 2017^24^ |  | ● | | ● |  |
| Wu et al., 2020^26^ | ● | ● | | ● |  |
| **Modification of cardiac electrophysiology** | | | | | |
| Shimizu et al., 1993^1^ |  |  | |  | ● |
| Kumagai et al., 2003^3^ |  | ● | |  |  |
| Goldstein et al., 2004^4^ |  | ● | |  | ● |
| Rossman et al., 2009^9^ |  | ● | | ● |  |
| Matsumoto et al., 2010^10^ |  | ● | |  | ● |
| Cakulev et al., 2011^12^ |  | ● | |  |  |
| Bhimani et al., 2014^16^ |  |  | |  | ● |
| Sadrpour et al., 2015^18^ |  | ● | |  | ● |
| Schwartzman et al., 2016^21^ | ● | ● | | ● |  |
| **Reduction of inflammation + rhythm control** | | | | | |
| Robinson et al., 2016^23^ | ● |  | | ● |  |
| **Substrate alteration** | | | | | |
| Becker et al., 2002^2^ |  | ● | | ○ |  |
| Yang et al., 2015^20^ | ● | ● | |  |  |
| **Other** | | | | | |
| Chang et al., 2018^25^ | ● | ● | |  |  |

† Studies reporting gross histopathological appearance of the atria without demonstrating statistical significance were classified as “Unclear”.

**Supplemental References**

1. Shimizu A, Kaibara M, Centurion O, Kapuku G, Hirata T, Fukatani M, Yano K, Hashiba K. Electrophysiologic effects of a new Class III antiarrhythmic agent, E-4031, on atrial flutter, atrial refractoriness, and conduction delay in a canine sterile pericarditis model. *J Cardiovasc Pharm.* 1993;21:656-662

2. Becker R, Senges JC, Bauer A, Schreiner KD, Voss F, Kuebler W, Schoels W. Suppression of atrial fibrillation by multisite and septal pacing in a novel experimental model. *Cardiovasc Res*. 2002;54(2):476-481. doi:10.1016/S0008-6363(02)00231-6

3. Kumagai K, Nakashima H, Gondo N, Saku K. Antiarrhythmic effects of JTV-519, a novel cardioprotective drug, on atrial fibrillation/flutter in a canine sterile pericarditis model. *J Cardiovasc Electrophysiol*. 2003;14(8):880-884. doi:10.1046/j.1540-8167.2003.03050.x

4. Goldstein RN, Khrestian C, Carlsson L, Waldo AL. AZD7009: A new antiarrhythmic drug with predominant effects on the atria effectively terminates and prevents reinduction of atrial fibrillation and flutter in the sterile pericarditis model. *J Cardiovasc Electrophysiol*. 2004;15(12):1444-1450. doi:10.1046/j.1540-8167.2004.04354.x

5. Kumagai K, Nakashima H, Saku K. The HMG-CoA reductase inhibitor atorvastatin prevents atrial fibrillation by inhibiting inflammation in a canine sterile pericarditis model. *Cardiovasc Res*. 2004;62(1):105-111. doi:10.1016/j.cardiores.2004.01.018

6. Ishii Y, Schuessler RB, Gaynor SL, Yamada K, Fu AS, Boineau JP, Damiano RJ Jr. Inflammation of atrium after cardiac surgery is associated with inhomogeneity of atrial conduction and atrial fibrillation. *Circulation*. 2005;111(22):2881-2888. doi:10.1161/CIRCULATIONAHA.104.475194

7. Tselentakis EV, Woodford E, Chandy J, Gaudette GR, Saltman AE. Inflammation effects on the electrical properties of atrial tissue and inducibility of postoperative atrial fibrillation. *J Surg Res*. 2006;135(1):68-75. doi:10.1016/j.jss.2006.03.024

8. Goldstein RN, Ryu K, Khrestian C, Van Wagoner DR, Waldo AL. Prednisone prevents inducible atrial flutter in the canine sterile pericarditis model. *J Cardiovasc Electrophysiol*. 2008;19(1):74-81. doi:10.1111/j.1540-8167.2007.00970.x

9. Rossman EI, Liu K, Morgan GA, Swillo RE, Krueger JA, Gardell SJ, Butera J, Gruver M, Kantrowitz J, Feldman HS, Petersen JS, Haugan K, Hennan JK. The gap junction modifier, GAP-134 [(2S,4R)-1-(2-aminoacetyl)-4-benzamido- pyrrolidine-2-carboxylic Acid], improves conduction and reduces atrial fibrillation/flutter in the canine sterile pericarditis model. *J Pharmacol Exp Ther*. 2009;329(3):1127-1133. doi:10.1124/jpet.108.150102

10. Matsumoto N, Khrestian CM, Ryu K, Lacerda AE, Brown AM, Waldo AL. Vanoxerine, a new drug for terminating atrial fibrillation and flutter. *J Cardiovasc Electrophysiol*. 2010;21(3):311-319. doi:10.1111/j.1540-8167.2009.01622.x

11. Yoo D, Vinten-Johansen J, Schmarkey LS, Whalen SP, Bone CC, Katzmark SL, Langberg J. Adhesive epicardial corticosteroids prevent postoperative atrial fibrillation. *Circ Arrhythm Electrophysiol*. 2010;3(5):505-510. doi:10.1161/CIRCEP.109.934299

12. Cakulev I, Lacerda AE, Khrestian CM, Ryu K, Brown AM, Waldo AL. Oral vanoxerine prevents reinduction of atrial tachyarrhythmias: Preliminary results. *J Cardiovasc Electrophysiol.* 2012;22(11):1266-1273. doi:10.1111/j.1540-8167.2011.02098.x.Oral

13. Mayyas F, Sakurai S, Ram R, Rennison JH, Hwang ES, Castel L, Lovano B, Brennan ML, Bibus D, Lands B, Barnard J, Chung MK, Van Wagoner DR. Dietary ω3 fatty acids modulate the substrate for post-operative atrial fibrillation in a canine cardiac surgery model. *Cardiovasc Res*. 2011;89(4):852-861. doi:10.1093/cvr/cvq380

14. Zhang Z, Zhang C, Wang H, Zhao J, Liu L, Lee J, He Y, Zheng Q. N-3 polyunsaturated fatty acids prevents atrial fibrillation by inhibiting inflammation in a canine sterile pericarditis model. *Int J Cardiol*. 2011;153(1):14-20. doi:10.1016/j.ijcard.2010.08.024

15. Schuessler RB, Ishii Y, Khagi Y, Diabagate K, Boineau JP, Damiano RJ. The effects of iflammation on heart rate and rhythm in a canine model of cardiac surgery. *Heart Rhythm*. 2012;9(3):432-439. doi:10.1016/j.hrthm.2011.09.074

16. Bhimani AA, Yasuda T, Sadrpour SA, Khrestian CM, Lee S, Zeng D, Belardinelli L, Waldo AL. Ranolazine terminates atrial flutter and fibrillation in a canine model. *Heart Rhythm*. 2014;11(9):1592-1599. doi:10.1016/j.hrthm.2014.05.038

17. Fu XX, Zhao N, Dong Q, Du LL, Chen XJ, Wu QF, Cheng X, Du YM, Liao YH. Interleukin-17A contributes to the development of post-operative atrial fibrillation by regulating inflammation and fibrosis in rats with sterile pericarditis. *Int J Mol Med*. 2015;36(1):83-92. doi:10.3892/ijmm.2015.2204

18. Sadrpour SA, Serhal M, Khrestian CM, Lee S, Fields T, Dittrich HC, Waldo AL. Termination of atrial flutter and fibrillation by k201’s metabolite m-ii: Studies in the canine sterile pericarditis model. *J Cardiovasc Pharmacol*. 2015;65(5):494-499. doi:10.1097/FJC.0000000000000219

19. Zhang Y, Wang YT, Shan ZL, Guo HY, Guan Y, Yuan HT. Role of inflammation in the initiation and maintenance of atrial fibrillation and the protective effect of atorvastatin in a goat model of aseptic pericarditis. *Mol Med Rep*. 2015;11(4):2615-2623. doi:10.3892/mmr.2014.3116

20. Yang G, Du X, Ni B, Chen H, Qi R, Cai C, Fang Y, Yang B, Ju W, Zhang F, Li M, Gu K, Shao Y, Chen M. Prevention of postsurgical atrial tachycardia with a modified right atrial free wall incision. *Heart Rhythm*. 2015;12(7):1611-1618. doi:10.1016/j.hrthm.2015.03.026

21. Schwartzman D, Badhwar V, Kormos RL, Smith JD, Campbell PG, Weiss LE. A plasma-based, Amiodarone-impregnated material decreases susceptibility to atrial fibrillation in a post-cardiac surgery model. *Innovations.* 2016;11(1):59-63.

22. Huang Z, Chen XJ, Qian C, Dong Q, Ding D, Wu QF, Li J, Wang HF, Li WH, Xie Q, Cheng X, Zhao N, Du YM, Liao YH. Signal transducer and activator of transcription 3/MicroRNA-21 feedback loop contributes to atrial fibrillation by promoting atrial fibrosis in a rat sterile pericarditis model. *Circ Arrhythm Electrophysiol*. 2016;9(7):1-13. doi:10.1161/CIRCEP.115.003396

23. Robinson E, Kaushal S, Alaboson J, et al. Combinatorial release of dexamethasone and amiodarone from a nano-structured parylene-C film to reduce perioperative inflammation and atrial fibrillation. *Nanoscale*. 2016;8(7):4267-4275. doi:10.1039/C5NR07456H

24. Ishii Y, Schuessler RB, Gaynor SL, Hames K, Damiano RJ. Postoperative atrial fibrillation: The role of the inflammatory response. *J Thorac Cardiovasc Surg*. 2017;153(6):1357-1365. doi:10.1016/j.jtcvs.2016.12.051

25. Chang CJ, Li SJ, Chen YC, Huang SY, Chen SA, Chen YJ. Histone deacetylase inhibition attenuates atrial arrhythmogenesis in sterile pericarditis. *Transl Res*. 2018;200:54-64. doi:10.1016/j.trsl.2018.06.002

26. Wu Q, Liu H, Liao J, et al. Colchicine prevents atrial fibrillation promotion by inhibiting IL-1β-induced IL-6 release and atrial fibrosis in the rat sterile pericarditis model. *Biomed Pharmacother*. 2020;129:110384. doi:10.1016/j.biopha.2020.110384
