# Supplementary material for: Systematic review of pre-clinical therapies for post-operative atrial fibrillation
Source: PLoS One. 2020 Nov 4;15(11):e0241643. doi: 10.1371/journal.pone.0241643 (PMC7641461; doi:10.1371/journal.pone.0241643)
Supplement: S2 File — (PDF) [file pone.0241643.s003.pdf]

Systematic review of pre-clinical therapies for post-operative atrial fibrillation

Darryl Davis, Chanhee Seo, Connor Michie, Benjamin Hibbert

Citation

Darryl Davis, Chanhee Seo, Connor Michie, Benjamin Hibbert. Systematic review of pre-clinical therapies for post-operative atrial fibrillation. PROSPERO 2019 CRD42019155649 Available from: https://www.crd.york.ac.uk/prospero/display_record.php?ID=CRD42019155649

Review question

We report the findings of all preclinical studies using animal experimentation to evaluate functional outcomes and summarize different approaches available to reduce post-operative atrial fibrillation.

Context and rationale

Post-operative atrial fibrillation (POAF) is a commonplace arrhythmia seen in a third of patients after coronary artery bypass grafting and almost half of patients after valve repair/replacement. Albeit often transient, the impact of POAF on surgical outcomes is significant as it portends a 2-fold increase in mortality, greater hospital resource utilization and increased costs. To date, a number of pharmacologic, dietary, and biologic agents have been studied in several preclinical models of POAF. Unfortunately, there remains a paucity in our understanding of an effective approach to prevent AF. In light of this, the purpose of this systematic review is to generate a critical appraisal of current literature on this issue while providing insight into the efficacy, plausibility and limitations of available prevention approaches. Herein, we report the findings of all preclinical studies using animal experimentation to evaluate functional outcomes and summarize different approaches available to reduce POAF.

Searches

The search strategies to be used for this review will be generated in collaboration with a Health Sciences Librarian with experience and expertise in designing systematic literature searches. A second information specialist with no association to the project will also review the strategy before executing the finalized search procedure. Validated preclinical search filters will also be applied to improve efficiency. There are no search restrictions for language or date of publication. We will search Ovid MEDLINE® and Embase Classic + Embase, In addition, a manual review of the bibliographies of included articles and relevant reviews will be performed. This search strategy will allow for a broad evaluation of the current literature and maximize the identification and inclusion of relevant data.

Study designs to be included

Inclusion criteria:

Both single- and double-arm studies will be included. Studies may be randomized, pseudo-randomized or non-randomized.

Exclusion criteria:

N/A

Human disease modelled

Post-operative atrial fibrillation

Animals/population

Inclusion criteria:

Pre-clinical in vivo models of postoperative pericarditis. These will include but are not limited to atriotomy, pericardiotomy and sterile pericarditis.

Exclusion criteria:

Ex vivo, in vitro, and invertebrate animal models will be excluded.

Intervention(s), exposure(s)

Inclusion criteria:

Any biologic, drug, procedure or dietary supplement done to reduce POAF.

All routes of intervention administration will be considered.

Experiments involving pre-or post-treatment.

Exclusion criteria:

Studies not investigating for therapeutic properties will be excluded (e.g. only investigated for their biodistribution).

Ablation and cardioversion studies will be excluded.

Comparator(s)/control

Inclusion criteria:

Studies with any comparator (e.g. vehicle control, sham-treated animals, no treatment, placebo, etc.) will be considered.

Exclusion criteria:

N/A

Outcome measure(s)

Inclusion criteria:

Atrial fibrosis is a important determinant of atrial fibrillation burden. We will include reported infarct size measured by any accepted method (i.e., histological or magnetic resonance imaging).

Atrial fibrillation inducibility is a standard electrophysiological measure done at the time of invasive EP study. It is reported as a binary outcome based on fulfilling a standard definition of 30 seconds.

Atrial fibrillation termination is a binary outcome observed following application of the therapy. All definitions of termination will be accepted.

Reduction in atrial fibrillation duration.

Atrial fibrillation duration is an important electrophysiologic marker for AF burden. It is typically reported as mean or median value. We will include both mean and median values in this review.

Exclusion criteria:

none

Study selection and data extraction

Procedure for study selection

Two reviewers will independently screen the titles and abstracts from the search results using the predefined inclusion criteria outlined above. A calibration test involving set of 10 studies will be performed to refine the screening question and ensure high inter-rated correlation (kappa >0.8), prior to formally commencing the screening process. For all titles/abstracts that appear to meet the inclusion criteria or where there is any uncertainty, we will obtain the full text article. Two reviewers will assess the eligibility of full-text articles. A calibration tests involving sets of 10 studies will be performed to ensure high inter-rater correlation. After every calibration test, the entire review team will be consulted to resolve any issues concerning full-text inclusion. After refining the full-text screening criteria, a formal screening process will be commenced. Any disagreement will be resolved through discussion with a third author. Reasons for study exclusion at this level will be recorded.

Prioritise the exclusion criteria

1) Not an original research publication (e.g. review article, editorial, comment)
2) Not an animal study

3) Not a therapeutic interventional study (e.g. the intervention was not directly administered as a therapy to animals) or only investigated for their biodistribution.

4) Induction of atrial fibrillation not using a postoperative model (e.g., electrical pacing only)

5) Ex vivo, in vitro, and invertebrate animal models will be excluded.
6) Ablation and cardioversion studies will be excluded.

Methods for data extraction

A standardized data extraction form will be designed by the review team to extract all relevant information from full-text articles. Finalized questions and answers will be uploaded onto DistillerSR. Information will be extracted independently and in duplicate from each eligible study. Calibration exercises using 3 studies will be conducted, and the review team will be consulted to refine the data extraction form. After commencing formal data abstraction, any disagreements will be first discussed between two independent reviewers. If no resolution can be made, a third-party team member will be consulted. The corresponding author of the individual studies included in our review will be contacted to obtain relevant missing data.

Data to be extracted: study design

Study characteristics:

- Sample randomization

- Study endpoint (when were the outcomes measured?)

- Single-arm vs. double-arm

- total number of animals used

- N per independent intervention group

- Sample size calculation

- Blinding of outcome analysis

Data to be extracted: animal model

Animal model (species, strain, sex, weight, mean, age, intercurrent illness of the animal)

Post-operative atrial fibrillation model (i.e., method to simulate POAF)

Data to be extracted: intervention of interest

Intervention characteristics:
- route of intervention delivery (intravenous, intracoronary, intramyocardial)
- timing of intervention
- frequency of intervention
- source of intervention (e.g. biological product species)

- vehicle

- any modification to therapy

- dose and units of dose used

Data to be extracted: primary outcome(s)

Primary outcomes

- Atrial fibrosis (% fibrosis area; continuous data)
- Atrial fibrillation inducibility (# of animals in which AF could be induced with, or without, treatment; dichotomous data)
- AF termination (# of animals in which AF was terminated after treatment; dichotomous data)

- AF duration (mean or median length, in seconds, of AF episodes induced; 25th-75th percentile will be extracted for data reported as medians; continuous data)

Data to be extracted: secondary outcome(s)

Secondary outcome - A qualitative summary of the putative mechanisms, adverse events, as well as retention and distribution of intervention in the model

Data to be extracted: other

N/A

Risk of bias and/or quality assessment

By use of SYRCLE’s risk of bias tool. By use of the CAMARADES checklist for study quality.

We will use Systematic Review Centre for Laboratory Animal Experimentation (SYRCLE’s) risk of bias tool, an adaptation of the Cochrane RoB tool specifically designed and validated for use in preclinical animal studies. This tool will assess selection bias (sequence generation, baseline characteristics, allocation concealment), performance bias (random housing, blinding), detection bias (blinding), attrition bias

(incomplete outcome data), reporting bias (selective outcome reporting), and other biases. Each criteria will be assigned a value of low, high or unclear risk of bias for each included study.

Risk of bias for each study will be assessed independently by two reviewers. Any discrepancies will be resolved by a senior author.

We will use the Collaborative Approach to Meta-Analysis and Review of Animal Data from Experimental Studies (CAMARADES) checklist to assess the quality of study data. Each study will be assigned a global quality assessment value of poor, average, or good quality as per the checklist. Each study will be assessed independently by two reviewers and any disagreements will be resolved by discussion and consensus.

.

Strategy for data synthesis

Planned approach

Categorical variables will be summarized by frequencies/percentages, and continuous variables will be summarized by means and standard deviations or median and interquartile ranges, depending on data distribution.

Studies will be pooled using Comprehensive Meta-Analyst (version 3; BioStat Inc., USA). Continuous endpoints will be pooled using standardized mean differences with inverse variance random effects modelling . If a critical mass of studies exist with similar methods of measurement (i.e., 4 independent studies)– mean differences will be used. Dichotomous endpoints (e.g. death) from each included study will be pooled and described as risk ratios and 95% confidence intervals. Results from outcomes with discrete data will be pooled and meta-analysis will be performed with inverse variance random effects modelling. Planned subgroup and sensitivity analyses will examine heterogeneity of the primary outcome. These will be carried out according to risk of bias assessments.

Effect measure

Continuous endpoints will be expressed as a standardized mean differences (SMD). SMD will be used given the expected heterogeneity in the method of measurement. Dichotomous endpoints (e.g. AF inducibility) will be expressed as risk ratios.

Effect models

Dichotomous outcomes will be analyzed using a random effects meta-analysis based on the DerSimonian Laird model and continuous outcomes will be analyzed using a random effects inverse variance meta-analysis.

Heterogeneity

Statistical heterogeneity of effect sizes will be assessed using the Cochrane I^2 statistic test. Thresholds for interpretation of I^2 are as follows: 0–40% (low heterogeneity), 30–60% (moderate heterogeneity), 50–90% (substantial heterogeneity), and 75–100% (considerable heterogeneity). If there is considerable heterogeneity (75–100%) then, sources of heterogeneity will be explored through subgroup and sensitivity analyses.

Other

We will correct for the multiple use of control groups using the method described by (Vesterinen et al., 2014).

Analysis of subgroups or subsets

Subgroup analyses

None planned

Sensitivity

None planned

Publication bias

None planned

Contact details for further information

Darryl Davis
ddavis@ottawaheart.ca

Organisational affiliation of the review

University of Ottawa Heart Institute

Review team members and their organisational affiliations

Dr Darryl Davis. University of Ottawa Heart Institute
Chanhee Seo. University of Ottawa Heart Institute
Connor Michie. University of Ottawa Heart Institute
Benjamin Hibbert. University of Ottawa Heart Insitute

Review type

Animal model review, Pre-clinical animal intervention review

Anticipated or actual start date

29 November 2019

Anticipated completion date

31 January 2020

Funding sources/sponsors

Canadian Institutes of Health Research

Conflicts of interest

None known

Language

English

Country

Canada

Stage of review

Review Ongoing

Subject index terms status

Subject indexing assigned by CRD

Subject index terms

Animals; Atrial Fibrillation; Postoperative Period

Date of registration in PROSPERO

03 December 2019

Date of first submission

05 November 2019

Details of any existing review of the same topic by the same authors

No

Stage of review at time of this submission

Stage	Started	Completed
Preliminary searches	Yes	No
Piloting of the study selection process	No	No
Formal screening of search results against eligibility criteria	No	No
Data extraction	No	No
Risk of bias (quality) assessment	No	No
Data analysis	No	No

The record owner confirms that the information they have supplied for this submission is accurate and complete and they understand that deliberate provision of inaccurate information or omission of data may be construed as scientific misconduct.

The record owner confirms that they will update the status of the review when it is completed and will add publication details in due course.

Versions

03 December 2019

PROSPERO
This information has been provided by the named contact for this review. CRD has accepted this information in good faith and registered the review in PROSPERO. The registrant confirms that the information supplied for this submission is accurate and complete. CRD bears no responsibility or liability for the content of this registration record, any associated files or external websites.
